# Evaluating changes and predictors of intention to act on health in urban development: a single-arm pre-post mixed-methods study of the changing mindsets intervention

**DOI:** 10.1186/s13690-026-01843-0

**Published:** 2026-02-06

**Authors:** Sophie L. Turnbull, Martha Jordan, Rebecca J. Linnett, Krista Bondy

**Affiliations:** 1 Bristol Medical School, University of Bristol, 39 Whatley Road, Bristol, BS8 2PS UK; 2 University of Bath School of Management, Convocation Avenue, Bath, BA2 7AZ UK; 3 Norwich Medical School, University of East Anglia, Norwich, UK; 4 Stirling Management School, University of Stirling, Stirling, FK9 4LA UK

**Keywords:** Urban health, Health equity, Psycho-social intervention, Chronic disease, Decision making

## Abstract

**Background:**

The built environment contributes to rising non-communicable diseases, including cancer, diabetes, respiratory illness, and poor mental health. These impacts disproportionately affect lower socio-economic groups, who experience greater exposure to harmful urban features. Evidence on these links alone has not led private-sector urban developers to prioritise health. The Changing Mindsets intervention was designed to increase intention to act on health and health inequalities among private-sector urban development professionals. This study assessed whether the intervention increased intention to act and identified predictors of change.

**Methods:**

We conducted a single-arm pre-post mixed-methods evaluation of the Changing Mindsets intervention delivered at six industry events in 2024, including four in-person and two online sessions. The intervention combined an industry-partner-led presentation, peer discussion, and a supporting website. Surveys were completed immediately before (T1), immediately after (T2), and three-months after the intervention (T3). Paired analyses assessed pre-post change in intention to act. Logistic regression examined predictors of improvement from T1 to T2. Seven participants completed follow-up interviews exploring changes in thinking and actions taken.

**Results:**

Of 156 attendees, 101 completed the T1 survey, 69 completed the T2 survey, and 22 completed the three-month follow-up. Immediately after the intervention, 19% of participants showed an increase in intention to act on health, while most showed no change. Paired analyses indicated a small increase in mean intention scores from T1 to T2, with limited evidence of a consistent group-level effect. Lower baseline intention and increased perceived psychological proximity to health issues were associated with improvement in intention to act. Mean intention scores remained elevated at three-months among respondents. Qualitative findings suggested that changes in thinking were linked to challenged assumptions about who can act on health. Reported actions most often involved initiating conversations and influencing others.

**Conclusions:**

The Changing Mindsets intervention was associated with increased intention to act on health among a subset of urban development professionals, particularly those with lower baseline intention and greater perceived proximity to health issues. Evidence for a strong average pre-post effect was limited. However, intention remained elevated at three-month follow-up among respondents, suggesting retention of motivation over time.

**Trial registration:**

ISRCTN12310546 registered on the 30th March 2021.

**Supplementary Information:**

The online version contains supplementary material available at 10.1186/s13690-026-01843-0.

**Table:** 

Text box 1. Contributions to the literature
• This study contributes to the literature in four key ways:
• Targets upstream determinants of health that are often excluded from public health interventions, by engaging private sector urban development decision-makers who shape population health and equity.
• Advances theory by evidencing psychological proximity as a precursor to action.
• Demonstrates a pragmatic, co-produced route to implementation of health interventions for private sector actors by embedding evidence and peer examples within industry-led events and resources.
• Offers a feasible mixed-methods approach to tracking early mechanisms and shorter term actions that precede measurable changes in population health.

## Background

The built environment has a significant impact on health and is contributing to the rise of non-communicable diseases (NCDs) such as respiratory illness, diabetes, cancer and poor mental health [1–3]. NCDs now account for 74% of all global deaths [4]. In the United Kingdom, NCDs have not risen evenly across the population. People living in deprived areas and from lower socio-economic groups experience higher rates of NCDs and spend more years of their lives in ill health [5–7]. These groups are also more likely to be exposed to harmful environmental conditions, such as higher levels of air and noise pollution, poor quality housing and extreme temperatures [8–11]. At the same time, they have reduced access to health-protective factors, including safe green spaces, healthy food shops and well-resourced health-services [11].

These unequal exposures have substantial societal and economic consequences. Air pollution alone is estimated to cost the UK £20 billion each year [12], while issues related to poor quality housing such as damp and mould cost the NHS an estimated £1.4 billion annually [13].

Health promoting features in the built environment can improve overall population health and tackle health inequalities [14–16]. For example, greater access to green space has been linked to lower prevalence of NCDs, including diabetes, depression, heart disease, with particularly strong benefits for those with lower socioeconomic status [16]. Residents of the greenest areas also show the smallest income-related health disparities [15, 16]. This highlights the significant potential for environments designed with health in mind to prevent illness and improve wellbeing.

Urban development, however, takes place within a complex system involving many stakeholders with competing priorities [17–20]. These stakeholders span national and local government, private sector organisations, community groups, the third sector and investors [17, 18]. In the UK, reductions in public sector capacity and ongoing cuts to public spending mean that private sector actors now have increasing influence over the design and delivery of urban environments [21–23]. This places private-sector professionals in a critical position to shape health outcomes.

Providing evidence about the health impacts of urban environments alone has not resulted in sustained increases in prioritisation of health within the private sector [19, 24]. As a result, there is a clear need for interventions specifically designed to shift the mindsets of private-sector professionals who influence urban development decisions.

The ‘Tackling Root Causes Upstream of Unhealthy Urban Development’ (TRUUD) programme mapped how the urban development system influences health and highlighted multiple points where intervention was possible [25]. The programme comprised of two phases. Phase 1 (2019–2022) drew on three main sources of evidence. First, a qualitative study with 132 public- and private-sector decision-makers showed broad agreement that health should be prioritised more in urban development [19]. Second, a systems map was developed through four multistakeholder workshops to identify how system elements connect to health outcomes [26].

These Phase 1 findings were synthesised to identify 50 possible intervention areas. These were refined to seven for development and evaluation in Phase 2 (2022–2025) [26, 27]. The selected interventions targeted leverage points across national government valuation mechanisms, city-region transport planning, law and health impact assessment, spatial planning, real estate investment, public engagement, and the private sector. This paper reports a mixed-methods evaluation of the Changing Mindsets intervention, examining its impact on intention to act on health and health inequalities among professionals working in the private sector of urban development, and identifying predictors of change. Intention to act was selected as the primary outcome. Intention to act is a construct used in behavioural psychology to describe the processes that precede action [28]. Intentions are understood as proximal goals that directly guide behaviour [28], and are therefore commonly used as a proxy for behaviour in behaviour change research [29].

## Methods

### Study design

We evaluated the Changing Mindsets intervention using a single-arm pre-post mixed-methods design implemented across six industry events in 2024. A single-arm pre-post design was selected because the intervention was delivered within existing industry events, which did not allow for a parallel control group or randomisation. The full study protocol, including methods and planned analyses, is available [30]. The process evaluation will be published separately to allow for the inclusion of sufficient detail, following the Medical Research Council (MRC) guidelines [31]. The Template for Intervention Description and Replication (TIDieR) checklist [32] was followed to ensure that appropriate details were included when reporting the findings of the study.

### Ethical and regulatory considerations

TRUUD programme received approval on 09/09/2020 from the University of Bristol Faculty of Health REC (ref: 94162). Amendment was received to include more details of this intervention development and evaluation on 05/01/2024 (ref: 6402). Participants received written and verbal information about the study and provided written informed consent prior to taking part in any survey or interview.

### Aims

The primary aims of this study were to explore:

1. Assess pre-post changes in intention to act on health from immediately before (T1) to immediately after the intervention (T2).
2. Examine whether contextual variables and theorised psycho-social influences (proximity, power, collective efficacy) predicted improvements in intention to act.

The secondary aims were to explore:

1. Explore longitudinal changes in intention to act and in the theorised psycho-social influences across T1, T2 and three-month follow-up (T3).
2. Examine whether participants reported any changes in their thinking about health three-months after the intervention.
3. Identify actions taken to prioritise health at the three-month follow-up.

### Study setting

#### Events

The intervention was evaluated at six stakeholder events, four in-person and two online between June and November 2024 (see Table 6: Event details and participant eligibility in the Additional file 1). The in-person events comprised two large urban development conferences and two smaller, invite-only sessions: one run in collaboration with the Housing Forum and the Town and Country Planning Association (TCPA), and another organized with the TRUUD team implementing an intervention with national government actors. The two online events included one developed with Women in Property and one hosted by the TRUUD team alone.

#### Participants

Eligibility criteria varied for each event (Table 6: Event details and participant eligibility in the Additional file 1). All sessions included professionals working in the private sector of urban development (referred to from here onward as the target group), alongside representatives from the third sector, local government and national government. Because these stakeholder groups operate within the same urban development system and influence one another’s decisions, we retained data from all attendees to reflect the real-world context in which the intervention is delivered and may exert impact. We also assessed whether belonging to the target group predicted improvement in intention to act and found no evidence of an association, indicating that inclusion of non-target participants did not materially affect the primary outcome. Where capacity was limited at the invite-only events, invitations prioritised mid- to senior-level decision-makers to maximise impact. Events were advertised through conference schedules, social media, partner organisation mailing lists, and TRUUD’s existing networks and newsletters.

### Changing mindsets intervention

#### Development of the theoretical framework to identify the influences of intention to act on health

A theoretical framework was developed to identify potential antecedents to intention to act on health that could potentially be triggered by our intervention. The potential antecedents were mapped by drawing on reviews of psychological and management literature, evidence and expert workshops. Subsequently four key psycho-social influences were identified due to the evidence of their influential impact on intention to act and their potential to be influenced by a behaviour change intervention. These were power, psychological proximity, collective efficacy and norms. Power was conceptualised as a psycho-social influence shaping individuals’ perceived ability to act in line with their intentions. Drawing on social and organisational theory, power was defined as comprising three interrelated forms. Resource-based power refers to access to material, financial, or informational resources that enable action [33]. Knowledge-based power reflects individuals’ understanding of how to act, their awareness of priorities, and their capacity to frame issues and influence discourse within their professional context [34–36]. Collective power captures the capacity to act alongside others, including the ability to influence norms and mobilise shared action [37]. These forms of power were selected because they are theoretically linked to intention formation and represent mechanisms that could plausibly be influenced through intervention.

Psychological proximity is about the closeness an event or concept feels to an individual [38]. In bringing events and problems more proximal to actors there is an increase in intention to act [39]. Psychological proximity is broken down into cognitive (relevance and salience) and emotional (connectedness and empathy) proximity that can both be used to shape intentions [39].

Collective efficacy is the combination of socio-cultural and cognitive influences that contribute to whether an individual believes that their group has the ability to achieve intended outcomes [40].

Norms refer to rules and standards that guide behaviour, including those held by individuals about their own conduct (personal norms) and those shared within a group or social context (social or group norms) [23].

#### Development and optimisation

The development, optimisation and content of the Changing Mindsets intervention is explored in depth elsewhere [41]. Briefly, the Changing Mindsets intervention was developed through an iterative co-production process using the Person-Based Approach and following MRC guidance [42–45]. The co-production involved a collaboration between the Changing Mindsets research team, expert advisors from the wider TRUUD team with a professional background in urban development, and two industry partners who are decision-makers within private sector urban development. These industry partners were representative of the target beneficiaries of the intervention. As such, they acted as target users within the co-production process to help ensure the intervention was relevant, acceptable and grounded in real-world private-sector practice.

Development and optimisation involved three stages: Stage 1 involved the collation of theory and evidence, which included the novel theoretical framework outlined above, primary mixed methods research (scoping search and semi-structured interviews with industry professionals) and stakeholder engagement [41]. Stage 2 translated these findings into guiding principles, behavioural-analysis tables and a logic model [41]. Stage 3 involved iterative intervention optimisation with the industry partners through ‘think aloud’ sessions [46, 47], multiple rounds of feedback via email and videocall, alongside event feedback. During implementation of the intervention, feedback from the participants and researcher field notes were reviewed following each event, any issues were recorded in the Table of Changes and prioritised by the team for modification. Changes were made where they improved the experience for the attendees or strengthened the messaging (e.g. making more time for discussion and removing detail from the slides). A follow-up meeting was arranged with each industry partner following their first event to discuss changes made to the intervention, to highlight sections where messaging could be strengthened, or to bring greater focus to intervention components such as the website or postcards. Further details of this process are reported in the forthcoming process evaluation.

#### Intervention design and core-components of the changing mindsets intervention

The Changing Mindsets intervention comprised a single facilitated workshop delivered in-person or online and supported by take-home and digital resources. The core components were consistent across all six events, with minor adaptations for delivery format (online or in-person). Industry partners co-produced a script for the presentation with the Changing Mindsets team, which contained the key talking points that they adapted to their own style and to include examples from their own experience. Recordings of an in-person and an online workshop are available here: https://www.truud.ac.uk/cmi-resources/. A copy of the presentation slides is available in Additional File 2: Example of Changing Mindsets presentation.

The workshop combined three main elements. First, participants received a structured presentation delivered by one of the two industry partners. This presentation introduced evidence on the links between urban development and health and health inequalities, including TRUUD-developed materials such as a short lived-experience film, findings from systems mapping, and the HAUS (Health Appraisal of Urban Systems) tool illustrating the health implications of features of the built environment. Industry partners also shared examples of how their own organisations and peers were acting to prioritise health into urban development decisions.

Second, participants took part in small group discussions. These discussions focused on reflecting on current practice, identifying barriers to prioritising health, and sharing examples of feasible actions within participants’ professional roles.

Third, participants were prompted to identify one or more concrete actions they could take following the session. At in-person events, this was supported through action-planning postcards; at online events, actions were recorded digitally. All participants were signposted to a dedicated intervention website via QR code or web link, which provided access to further evidence, tools, case studies and networks to support ongoing action.

While delivery style and examples varied slightly depending on the industry partner and event context, the intervention content, behaviour change targets, and core structure remained consistent across events.

### Outcomes

The primary outcome was change in intention to act from T1 to T2, which was measured using a single item adapted from an existing scale of willingness to act, with slight wording amendments [48]. This was “I intend to take concrete steps to do something to integrate health into my work”. Further details of item development and scale construction are provided in the published study protocol [30].

The secondary outcomes were change in intention and in theorised psycho-social influences (proximity, collective efficacy, perceived power, norms) across three time-points (T1, T2, three-months following the intervention (T3)), and self-reported change in thinking and actions on health three-months after the intervention.

- Psychological proximity was measured using five items from an existing measure of proximity [39, 49]. Three items were designed to tap into cognitive proximity and two into emotional proximity and included statements such as “This issue needs to be addressed”. None of the items were reverse-scored and higher scores indicated greater psychological proximity. Internal consistency reliability for the full scale was 0.54/0.75/0.70 across the three timepoints.
- Collective efficacy was measured using nine items in total. Three were from an existing scale designed to tap into the subdimension of empowerment, with slight wording amendments [50], three were from an existing scale designed to tap into the subdimension of social cohesion, with slight wording amendments [51, 52], and three were developed for the purpose of this study to tap into the subdimension of social control [30]. Items included statements such as “The organisation I work for is effective in achieving its goals”. None of the items were reverse-scored and higher scores indicated greater collective efficacy. Internal consistency reliability for the full scale was 0.88/0.87/0.82 across the three timepoints.
- Power was measured using six items in total; three were from an existing scale designed to tap into the subdimension of resource-based power, with slight wording amendments [53], and three were developed for the purpose of this study to tap into the subdimension of knowledge-based power [30]. Items included statements such as “I have a good understanding of how my work can impact health via the built environment”. One of the items was reverse-scored and higher scores indicated a greater sense of power. Internal consistency reliability for the full scale was 0.77/0.75/0.83 across the three timepoints.
- Group norms were measured using 12 items in total, developed for the purpose of this study based on prevalent norms within the industry sourced from pilot interviews with private sector urban development professionals [30]. The 12 items consisted of six pairs of descriptive and injunctive norm statements that focused on different aspects of pro-health norms within urban development and included statements such as “It is acceptable in my industry to treat health as a low priority”. Eleven items were reverse-scored and higher scores indicated greater endorsement of pro-health industry norms. Group norms were not measured immediately after the intervention at Timepoint 2 as it was felt that any changes would take longer to develop. Internal consistency reliability for the full scale was 0.84/0.89 across the two timepoints.

### Data collection

At each event, attendees received an information sheet, provided written consent, and completed a baseline survey at T1, which measured our theorised psycho-social influences and collected sociodemographic data. Full data collected at each timepoint is provided in Additional file 3 Table 7: Data collected across the three timepoints. Sessions were audio-recorded to capture facilitator delivery, with breakout discussions kept confidential and not recorded. Immediately after the intervention, participants completed the T2 survey assessing changes in intention to act, the targeted psycho-social influences, fidelity of delivery, and their experience of the session. Paper‐and‐pencil surveys were used in-person and online surveys for virtual events. Members of the research team observed the session and took field notes, and photographed paper and online feedback from the discussion session to record discussions and feedback given in the session. Three-months after the intervention session, participants were emailed a link to the T3 survey that reassessed intention to act, the psycho-social influences and asked about any actions taken to prioritise health in their work. Engagement with intervention components was explored through attendance at the event session, field notes and recordings of engagement and discussion at the event, use of the website, and through free-text responses from the surveys and direct questioning in follow-up interviews.

### Analysis

#### Quantitative data analysis

We summarised participant characteristics at each time point using medians, interquartile ranges and ranges. The ’target group’ variable was generated from industry and role from the T1 surveys; categorisation of these data are provided in Additional file 4 Table 8: Categorisation of industry and occupation into target group. Likert items were treated as continuous for descriptive analyses, consistent with evidence supporting parametric treatment of such data [54].

To assess pre-post change in intention to act from T1 to T2, we used paired analyses treating intention scores as continuous. Because intention scores showed non-normal distributions and ceiling effects, we conducted a Wilcoxon signed-rank test. As a sensitivity analysis, we also ran a paired t-test to describe the mean change and corresponding confidence interval. These paired tests were used to quantify the direction and magnitude of change rather than to make dichotomous claims about effectiveness.

To examine predictors of improvement in intention to act, we operationalised improvement as a binary indicator of any increase in intention from T1 to T2 (increase vs. no increase). This dichotomised outcome was used to explore heterogeneity in response and align with the predictor analysis, rather than to assess overall intervention effectiveness. Logistic regression was selected due to the non-normal distribution of intention scores and ceiling effects. Missing data were handled using complete case analysis, whereby only participants with full data for the variables included in each model were analysed. Because attrition may not have been random, resulting estimates may be biased and should be interpreted cautiously.

Our primary outcome was change in intention to act from T1 to T2; we did not model T3 due to its small sample size (*n* = 22). To avoid overfitting given our T2 sample size (*n* = 64), we limited the multivariable model predictors to approximately 10% of observations. Predictor selection was guided by theory, drawing from a longer list defined a priori in the study protocol [30]. This included sociodemographic characteristics (age, gender, target group) that can impact power, and the theorised psycho-social influences (baseline intention to act as a continuous variable, and improvement in proximity, power, and collective efficacy) that directly addressed our theorised pathways to change in intention to act [30]. This directly addresses Type I error concerns without the need for stringent alpha adjustments, which would unduly reduce power and increase Type II error risks in our relatively small sample [55, 56]. Norms were not included in this model as we did not have data at T2. The full logistic regression model was fitted by entering all variables, then variables with weaker evidence of association (*p* > 0.05 and wide CIs) were sequentially removed. We compared nested models via likelihood-ratio tests, pseudo-R² and Akaike’s Information Criterion to confirm improved fit [57]. As this was an exploratory study, we reported precise p-values and included trends that reached *p* < 0.10 regardless of whether it reached the traditional *p* < 0.05 threshold, and details were included in the text [58].

For our secondary analysis of change across T1, T2 and T3, we again treated Likert items as continuous to describe trajectories over time. We explored mean changes in both available cases and complete cases to assess the robustness of our temporal trends and the potential impact of attrition on the observed trajectories [59].

#### Qualitative data analysis

Three-months after attending the intervention session, seven intervention participants (of the 74 invited) consented to take part in the qualitative interviews. Verbal and written informed consent was collected prior to the interview. Interviews lasted between 43 and 73 min, were conducted by videocall and recorded on an encrypted audio recorder. The recordings were then transferred to the University of Bristol secure servers. They were transcribed and uploaded to NVivo (Version 1.6.2; Lumivero) for analysis [60].

The interviews were semi-structured following a topic guide, which is available in the Additional file 5. The topic guide sought to explore any action they had taken to integrate health into their work, their views on the intervention, to investigate the proposed mediators of intention to act (collective efficacy, group norms, proximity, power), and to identify any wider impacts for the intervention (e.g. other individuals or organisations that they believe they have impacted as a result of the intervention).

The interviews were conducted and coded by two team members (MJ, RL). Thematic analysis was iterative and abductive [61], with deductive codes identified prior to the start of data analysis and inductive codes identified through immersion in the data [61]. Initial codes were developed by RL and MJ, who met to discuss emerging codes and the developing code hierarchy to improve shared understanding and consistent coding. The whole multidisciplinary team (MJ, RL, KB and ST) met to discuss the patterns of meaning (potential themes). MJ and ST organised the codes into final themes, which were agreed upon by the team.

#### Combining qualitative and quantitative data

The quantitative and qualitative analyses were used to build on each other; qualitative data was used to explain quantitative findings.

## Results

### Events

The approximate sample who attended the 6 events was *n* = 156, with some attendees arriving late and leaving early in the webinars and events with a conference format where there were parallel sessions available (Table 6: Event details and participant eligibility in the Additional file 1). The median number of attendees across the 6 events was 21 (range 8–63).

### Samples

#### Survey

Of the 156 attendees, 101 completed the baseline survey before the intervention (two partially completed), 69 completed the follow-up survey (four partially completed) and 22 participants completed three-month follow-up data. Of the 22 participants who completed three-month follow-up, there were five participants who completed baseline and three-month follow-up only.

There were sociodemographic data for *n* = 97/101 (96.0%) participants who provided T1 data, 67/69 (97.1%) of those who provided T1 and T2 data, and *n* = 22/22 (100.0%) who provided baseline and three-month follow-up (T3) (Table 1). Sociodemographic characteristics of the samples were largely consistent across time-points. However there were changes in proportions over time for gender and age, ethnicity and target group oscillated over the time-points, and years in role remained stable. The proportion of male participants increased slightly across time-points (25.8% at T1, 29.9% at T2, and 40.9% at T3). The median age also increased slightly across time-points: T1 39.5 years (IQR: 31–48, Range: 23–74), T2 41 years (IQR: 32–51, Range: 23–74), T3 43.5 years (IQR: 31–54, Range: 23–71). The proportion of the target group represented in the sample at each time point remained relatively stable (76.3% at T1, 74.2% at T2, and 77.3%). Participants’ median years in role also remained stable across time-points: T1 5 years (IQR: 2–17, Range: 0.08–46), T2 5 years (IQR: 2–18, Range: 0.08–46), T3 5 years (IQR: 1–21, Range: 1–40). Ethnicity representation varied slightly across time-points, with white British participants being represented in greater proportions at T2 (50.8%) compared to T1 (42.3%), and T3 (45.4%). The smaller sample size of participants from other ethnic backgrounds, made patterns more difficult to interpret.

As the study did not collect individual-level reasons for attrition, we cannot report specific causes of withdrawal. Follow-up completion relied on participants responding to email invitations after the event, which may have contributed to non-response. Sociodemographic characteristics were broadly similar across time-points, offering some reassurance that attrition did not substantially shift the composition of the sample.

**Table 1:** Sociodemographic characteristics of participants completing surveys across the three time-points

Sociodemographic Variable		Immediately before the intervention (T1) (*n*/*N* (%)) or Median (IQR, Range)	Immediately after the intervention (T2) (*n*/*N* (%) or Median (IQR, Range))	Three-Month Follow-up (T3) (*n*/*N* (%) or Median (IQR, Range))
Gender	Female	72/97 (74.2%)	47/65 (70.2%)	13/22 (59.1%)
	Male	25/97 (25.8%)	20/65 (29.9%)	9/22 (40.9%)
Target group	Yes	74/97 (76.3%)	49/65 (75.4%)	17/22 (77.3%)
	No	22/97 (22.7%)	17/65 (26.2%)	5/22 (22.7%)
Ethnicity	White British	41/97 (42.3%)	33/65 (50.8%)	10/22 (45.5%)
	White - Other	24/97 (24.7%)	14/65 (21.5%)	9/22 (40.9%)
	Mixed background	4/97 (4.1%)	2/65 (3.1%)	1/22 (4.6%)
	Black British/African	2/97 (2.1%)	1/65 (1.5%)	1/22 (4.6%)
	Asian	2/97 (2.1%)	2/65 (3.1%)	0/22 (0.0%)
	Other/Not Stated	24/97 (24.7%)	13/65 (20.0%)	1/22 (4.6%)
Age	Years	39.5 (IQR: 31–48, Range: 23–74)	41 (IQR: 32–51, Range: 23–74)	43.5 (IQR: 31–54, Range: 23–71)
Years in Role	Years	5 (IQR: 2–17, Range: 0.08–46.08)	5 (IQR: 2–18, Range: 0.08–46.08)	5 (IQR: 1–21, Range: 1–40)

#### Interviews

Of the seven (four male, three female) participants who took part in the three-month follow-up interviews, all identified as white/white British, with ages ranging from 31 to 71 years (mean 54.7). Four of the participants were from the target group and three were not. Their professional backgrounds included urban design/public health, built environment, landscape architecture, planning and development, academia/NHS, and civil engineering, with years of experience ranging from one to over forty (mean 20). We interviewed at least one person from each event, for two of the events two people were interviewed.

### Primary aims

#### Pre-post effectiveness of the intervention from T1 to T2

Of the 69 people who provided T1 and T2 data, there were 64 participants where there was intention to act data for both time-points. For the majority of participants, intention to act on health did not change (70.4%, *n* = 45/64), a positive shift occurred in *n* = 12/64 (18.7%) participants, and a negative shift for *n* = 7/64 (11.0%) (Table 2). The baseline intention to act was high across the participants, with no one disagreeing or strongly disagreeing that they intended to act on health. Increases in intention to act were observed primarily among those starting at lower baseline levels (“Neutral” and “Agree”), while decreases were rare and limited to participants starting at “Agree” and “Strongly Agree.” (Table 2).

Paired analyses suggested a small upward shift in intention to act from T1 to T2, but there was limited evidence of a consistent change across the sample as a whole. The Wilcoxon signed-rank test indicated only weak evidence for a systematic pre-post shift (z = 1.15, exact *p* = 0.30), which is consistent with the high baseline intention scores and resulting ceiling effects. The paired t-test showed a mean increase of 0.09 points on a five-point scale (95% CI − 0.07 to 0.26), indicating that the average change was small and estimated with considerable uncertainty.

**Table 2:** Change in intention to act from baseline (T1) to immediately following the intervention (T2)

Baseline intention to act (T1)	Change in agreement intention to act from T1 to T2				
	Decreased by 2	Decreased by 1	Did not change	Increased by 1	Increased by 2
3 (Neutral)	0	0	4	4	2
4 (Agree)	0	5	23	6	NA
5 (Strongly agree)	1	1	18	NA	NA
Total	1	6	45	10	2

#### Predictors of improvement in intention to act

##### Model ascertainment

The full and final models are available in Table 3. The full model included 62 participants due to missing sociodemographic data for two cases. There was no evidence of an association between any of the sociodemographic characteristics, or power and improvement in intention to act. There was strong evidence of a negative association between baseline intention to act and improvement (β = −2.83, *p* = 0.007, 95% CI: −4.90, −0.76), and evidence of a positive association between proximity and improvement in intention to act (β = 2.46, *p* = 0.05, 95% CI: 0.03, 4.88). There was also a trend indicating an inverse relationship with collective efficacy (β=−1.96, *p* = 0.10, CI:−4.30, 0.38).

##### Final model

To fit the final model, variables were iteratively removed starting with the weakest evidence of an association. In the final logistic regression model (*n* = 64), the overall fit was supported by a likelihood ratio χ² of 25.86 (*p* < 0.001) and a pseudo R² of 0.42, indicating that the predictors collectively accounted for approximately 42% of the variance in the outcome (Table 3). Improvement in proximity was associated with a substantial increase in intention to act (β = 2.45, *p* = 0.037; 95% CI: 0.15, 4.75). In contrast, there was a robust inverse association between baseline intention to act and improvement in intention to act at T2 (β = − 2.60, *p* = 0.006; 95% CI: − 4.45, − 0.76), indicating that higher initial intention was linked to lower odds of further increases in intention to act. In the full model, collective efficacy remained negatively associated with improvement in intention to act, but the association was weaker and did not reach conventional levels of statistical significance (β = −1.84, *p* = 0.11, 95% CI: −4.08 to 0.39). Power was also included in the model due to the theoretical importance of the variable and because model fit statistics suggested only a minimal improvement in model parsimony where power was not included (AIC 45.91 vs. 45.15 and BIC 56.71 vs. 53.79). There was no evidence of an association between improvement in power and improvement in intention (β = 0.94, *p* = 0.27, 95% CI: −0.75, 2.64).

**Table 3:** Full and final model of predictors of improvement in intention to act

Predictor	Increase in intention to act (*n*/*N*, %)	No increase in intention to act (*n*/*N*, %)	Full model (*n* = 62): Coefficient, *p*-value, 95% Confidence Interval [CI]	Final model (*n* = 64): Coefficient, *p*-value, 95% Confidence Interval [CI]
Gender (Male)	4/12 (33.3)	14/51 (27.5)	β 0.06, *p* = 0.95, [−1.73, 1.85]	Not in the final model
Target Group (yes)	10/12 (83.3)	36/50 (72.0)	β 1.33, *p* = 0.37, [−1.58, 4.23]	Not in the final model
Improvement in Proximity to the Issue (yes)	11/12 (91.7)	24/52 (46.2)	β 2.46, *p* = 0.05, [0.03, 4.88]	β = 2.45, *p* = 0.037, [0.14, 4.75]
Improvement in Collective Efficacy (yes)	2/12 (16.7)	18/52 (34.6)	β −1.96, *p* = 0.10, [−4.30, 0.38]	β= −1.84, *p* = 0.11, [−4.08, 0.39]
Improvement in Sense of Power (yes)	7/12 (58.3)	23/52 (44.2)	β 0.67, *p* = 0.46, [−1.11, 2.44]	β = 0.94, *p* = 0.27, [−0.75, 2.64]
	**Median ** **(Range, Interquartile range)**	**Median (Range, Interquartile range)**		
Age (Years)	32 (26–53.5)	43 (35–51)	β −0.01, *p* = 0.57, [−0.08, 0.04]	Not in the final model
Baseline Intention to Act	3.5 (3–4)	4 (3–5)	β −2.83, *p* = 0.007, [−4.90, −0.76]	β= −2.60, *p* = 0.006, [−4.45, −0.76]

### Secondary aims

#### Longitudinal changes in intention to act and theorised psycho-social influences

Descriptive statistics for intention to act, and our theorised psycho-social influences are shown in Table 4, with means calculated both on all available cases (T1 *n* = 98, T2 *n* = 65, T3 *n* = 22) and on the 17 participants who provided data at all three survey timepoints. Using all available cases, intention to act rose from a T1 mean of 3.95, to 4.25 at T2 and remained elevated at 4.23 by T3. Perceived proximity increased from 4.07 (SD 0.59) at T1 to 4.35 (SD 0.47) at T2 and held at 4.13 (SD 0.56) at T3. Perceived power showed a small increase from 3.70 (SD 0.63) at baseline to 3.85 (SD 0.54) post-intervention before returning to 3.74 (SD 0.57) at follow-up. Pro-health norms declined modestly from 2.94 (SD 0.58) at T1 to 2.80 (SD 0.67) at T3. For intention to act, proximity, power, and pro-health norms the complete case data followed the same trajectory as the full case. In the full case data collective efficacy was stable at a mean of 4.00 (SD 0.53–0.56) through T1 and T2 and rose to 4.06 (SD 0.49) at T3; while the complete case data started higher (4.14) then dropped at T2 (4.02) and increased slightly at T3 (4.06). The near-identical trajectories observed in both the full-case and complete-case analyses indicate that participant attrition did not materially bias our descriptive findings for change over time.

**Table 4:** Median and mean scores for main study variables across three timepoints

Theorised psycho-social influences of intention to act	Survey timepoint		
	Baseline- T1	Immediately after the intervention- T2	Three-months following the intervention- T3
Intention to act			
Mean available cases (SD)	3.95 (0.80)	4.25 (0.71)	4.23 (0.81)
Mean complete case (SD)	4.11 (0.60)	4.35 (0.60)	4.24 (0.90)
Proximity (mean of scale items)			
Mean available cases (SD)	4.07 (0.59)	4.35 (0.47)	4.13 (0.56)
Mean complete cases (SD)	4.11 (0.56)	4.48 (0.39)	4.25 (0.41)
Collective efficacy (mean of scale items)			
Mean available cases (SD)	4.00 (0.53)	4.00 (0.56)	4.06 (0.49)
Mean complete cases (SD)	4.14 (0.54)	4.02 (0.57)	4.06 (0.45)
Power (mean of scale items)			
Mean available cases (SD)	3.70 (0.63)	3.85 (0.54)	3.74 (0.57)
Mean complete cases (SD)	3.82 (0.46)	3.94 (0.52)	3.77 (0.65)
Norms (mean of scale items)			
Mean available cases (SD)	2.94 (0.58)	-	2.80 (0.67)
Mean complete cases (SD)	2.91 (0.66)	-	2.80 (0.67)

#### Changes in thinking about health

The interviews undertaken at T3 provided insight into changes in thinking about health following the intervention, where a lack of change was associated with a strong prior interest in health, and change was associated with having their assumptions challenged about who acts on health.

##### Affirming and reinforcing practice

Many participants explained that they were already prioritising health in their professional roles before the intervention. For these participants the intervention session affirmed existing views or provided space for reflection on practice rather than transforming their thinking about health. This supports the quantitative findings that baseline intention to act on health was high, and that those with a higher baseline intention to act were less likely to demonstrate improvement following the intervention. As one participant put it *“I was already bought in to that (…) I was already doing it”* (ID 47, not target group). Others described the session as *“reinforcing what we do (…) how we do it*,* and why…”* (ID P26, not target group) or it *“affirmed… things that we knew intuitively”* (ID 28, target group). A few described the session as prompting reflection on different aspects of their work, with one participant commenting that it *“got [them] to think about different things [they’re] doing”* (ID 101, not target group).

##### Challenging assumptions

Two participants described how the industry partner delivering the intervention, views of other attendees and presentation data challenged their views about who is willing or able to prioritise health. One reflected on how their previous professional experience had led them to view developers as reluctant to engage in health, and expressed surprise that industry professionals were involved in delivering the intervention:

*“I think I was quite surprised by that talk… it didn’t reflect what I’d heard from housing developers… So it was quite interesting to hear that there were people who were interested in that… I guess I didn’t understand how you managed to get corporate funding to support it. Like*,* what’s in it for them…”* (ID 47, not target group, event 3).

Another participant who had been working in the field *“since the late 70s”* described having their *“bias”* challenged by discussion with other workshop attendees who were early career professionals. They reflected on how this prompted them to reconsider their views on the challenges of prioritising health in urban development: *“I’d been working on some old statistics”* (ID 26, not target group, event 1).

##### Reflecting on intervention influence

During the interviews, we asked the participants to reflect on why intention to act on health may have increased for some and decreased for others after the intervention. Participants speculated about what they thought may have influenced changes in intention to act, although none reported feeling that their intention to act had decreased. One participant suggested that increases in intention could be linked to gaining new awareness ‘*of the impact of housing on health and they had learnt something*,* and maybe they’ve recognised that they’re in a position to make change*” (ID 47).

In terms of possible reasons for decreases in intention to act, some participants suggested it may be related to the intervention content or variation in delivery. One suggested that attendees may have expected clear, actionable guidance, rather than the open-ended, reflective format of the session. Suggesting attendees may have been “*hoping for ‘do these five things in this order please*,* and you’ll have a utopian life*’” (ID 39). Another participant considered whether variation in workshop delivery or having a negative experience might have influenced responses: “*…the workshop I was in seemed fine to me (…) I don’t know whether this is true*,* but I know some of these workshop events (…) people can come away with wildly different impressions of a day*,* depending on that workshop(…) I’m surprised people said that it [intention to act] went down…*” (ID 101).

External constraints were also raised as a potential influence, with one participant commenting that cost and time pressures may have limited participants’ perception of their ability to act: “*the appetite is very much focused on just cost and time efficiency (…) people are finding it more difficult to kind of make these changes*,* and they might see themselves doing it less or more neutral*” (ID 72).

Some participants questioned whether changes in intention reflected real shifts or were not “*an intentional direction*” (ID 47), but instead an artifact of the data collection process. One said: “*when you fill out a survey and (…) you can’t remember what you put before and probably try to put the same*” (ID 47). Another reflected on the effect of survey length and response fatigue: “*they get to like halfway through and then start going*,* oh just agree*,* agree*,* agree… they’re busy*,* oh right let’s get this through*,* let’s get it done… they don’t always remember what they’ve put the first time round*” (ID 101).

### Actions taken on health 

We explored actions taken by the participants through data from interviews and surveys at three-months following the intervention.

#### Actions taken to prioritise health described in the interviews

Interview participants described a range of actions taken following the intervention, including: increased discussion about health, using tools and information from the intervention to convince others to act on health, and undertaking specific activities to prioritise health. They also reflected on reasons why there was an intention-action gap for some people.

##### Convincing others

In agreement with the surveys, participants most frequently described taking action by initiating conversations with others about health. Some spoke about having positive reactions from colleagues when these discussions occurred: *“They’re always supportive*,* because what we all do*,* is we assimilate quite well. It may take us a while*,* but we assimilate quite well”* (ID 28, target group). The same individual reflected on the shared commitment to making positive environments by private sector professionals they worked with: *“the majority of house builders that we would work with have got a much higher level of*,* you know*,* they want to make a good place so they’re not trying to cut corners everywhere along the line”* (ID 28). In contrast, another participant described barriers to engaging others: *“I have slight problems in getting our corporate teams to understand things sometimes”* (ID 101, not target group).

Many of the interviewees displayed a willingness to encourage others to engage with health in their work: “*we’re trying to do a lot of influencing across as many spheres as we can”* (ID 101, not target group). For one person, interactions in the workshop made them aware that many people did not know what Joint Strategic Needs Assessments (JSNAs) were, prompting them to do *‘…quite a bit of work…around sort of getting that message [about JSNAs] out to our local authorities…[and] developers.’* (ID 101, not target group). Another said they spoke to their manager about wanting to focus on work related to physical environments and upstream determinants of health.

One participant spoke about how they were developing networks of those interested in health and discovering existing expertise in the organisation they would be able to draw on in the future: *‘…the more I talk to some of my colleagues*,* (…)…they’re like*,* best practice [on health] is to do x*,* y*,* z. Yeah*,* that was really interesting to me is how much our colleagues are already doing quite a lot of this and I just wasn’t aware of it.’* (ID 72, target group).

##### Shared learning and persuasion

Some participants described how they used the tools and data from the Changing Mindsets intervention to support their discussions about health and persuade colleagues or external stakeholders to act on health. One participant forwarded the website to others, reflecting that *‘ it’s been a very useful tool to (…) say ‘and here is the evidence*,* go look…’* (ID 28, target group). Another referred to using the data from the presentation in their work: *“you’ve given us some data*,* which we’re obviously able to use. I’m not going to say how the others have used it*,* but I know they have”* (ID 26, not target group).

##### Taking action

Participants also described specific activities that they had undertaken which were prompted by attending the intervention. One participant *‘tried to raise funds internally’* to update a paper to *‘incorporate a way of pricing (…) the mitigation of long term chronic illness’* (ID 39, target group). Another spoke about how they found the lived experience of the noise pollution in the video really powerful, taking the issue from an ‘*abstract concept’* to ‘*understanding just how much that does impact your living environment as well*,* and can be really miserable’* (ID P72, target group). This participant then described raising the issue of noise attenuation in their projects and *“pushing it up the agenda a little bit.”*

##### Intention- action gap

Despite having set intentions to act, a number of participants acknowledged that they had not yet followed through due to conflicting priorities, or feeling like it was too big a project to take on at the time. One participant said their recorded action: *“…might have been about like connecting with other people from the webinar*,* and I don’t actually know if I did that (…) I think it just sort of slipped off the… you know*,* the back of the table kind of thing. I think I did connect with a couple of them*,* but the conversations have not continued*.*”* (ID 72, target group). Another noted that they were able to achieve some of the actions they had planned that were lighter touch, but other planned actions needed more time and resources to undertake: ‘*I think mine [action] was (…)talking to people about TRUUD and about*,* talking about health more (…) and I definitely have been doing that. I don’t – I also wrote something down which was completely separate (…) and I haven’t done this ‘cause it’s a whole blimming project…*’ (ID 28).

#### Actions taken reported in the surveys

The median actions taken by participants was 2 (IQR 1–3, range 0–7), and 4 did not report taking any actions. Table 5 illustrates the number of actions taken by the participants to integrate health into their work at T3.

**Table 5:** Number of actions taken by the participants at three-month follow-up

Number of actions taken	*n*/*N*	%
0	4/22	18.18
1	3/22	13.64
2	8/22	36.36
3	3/22	13.64
5	1/22	4.55
6	2/22	9.09
7	1/22	4.55

**Fig. 1:**
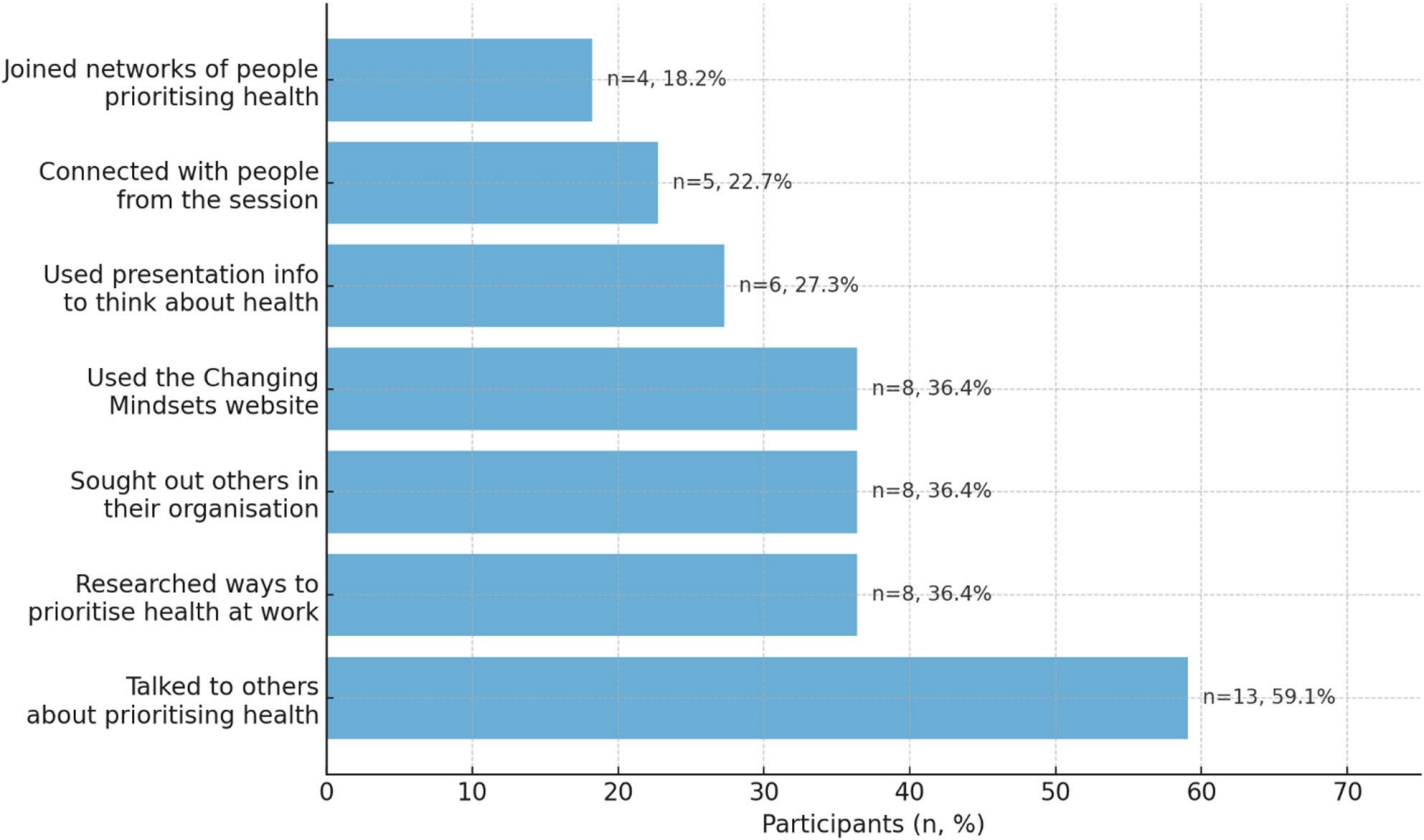
Actions taken at three-months following the intervention

Figure 1 shows the types of actions taken by participants at the T3 survey (*n* = 22). The most frequently reported action was having conversations with others about prioritising health (*n* = 13/22, 59.1%), followed by 36.4% (*n* = 8/22) who had either researched ways to prioritise health in their work, sought out others in their organisation who were thinking about or acting on health, or used the Changing Mindsets website. A further 27.3% (*n* = 6/22) participants reported that they used information given in the presentation to think about health in their work, 22.7% (*n* = 5/22) connected with people they met in the session, and 18.2% (*n* = 4/22) joined networks of people prioritising health. These findings were reinforced by the qualitative interviews.

Pre-intervention actions were not assessed at baseline (T1), so the actions reported at three-month follow-up reflect participants’ perceptions of what they had done during the post-intervention period rather than a measured change from baseline. As such, these actions cannot be attributed solely to the intervention and may also reflect wider ongoing practice.

## Discussion

### Summary of main findings

Immediately after attending the intervention, approximately one fifth of participants showed an increase in intention to act on health, while most participants’ intention remained stable. Paired analyses indicated a small upward shift in intention scores at the group level. However, p-values and wide confidence intervals suggest limited evidence for a strong overall change, reflecting both ceiling effects and high baseline motivation among attendees. These findings indicate that the intervention was more effective in shifting intention among a subset of participants rather than producing a large average change across the whole sample.

The inverse association between baseline intention and improvement further supports this interpretation, suggesting that participants who already reported high intention to act had less scope for measurable improvement. This pattern is consistent with prior research showing that intention is more likely to change among those starting from lower baseline levels, while high initial intention tends to be stable over time [62, 63].

Our study also confirms previous findings that an increased sense of proximity to the issue leads to increased intention to take pro-environmental actions, such as recycling and supporting an online social media recycling campaign [39, 49].

Our secondary outcomes explored mean changes in intention to act and the theorised psycho-social influences of intention to act across the three time-points, and changes in thinking and actions taken at three-months. Intention to act rose immediately after the intervention and remained higher at three-month follow-up, with only a 0.02 point decline, suggesting the intervention had a lasting influence on participants’ intention to take action.

Perceived proximity and power also increased post-intervention but returned near baseline by three-months, perhaps reflecting an initial boost in agency and closeness to the issue that waned when faced with systemic constraints or a lack of ongoing support [64–66]. The small decline in perceived pro-health norms from baseline to three-months, may reflect the challenge of shifting entrenched cultural norms over a short period [67], with a single session of the intervention, and without cultural change within the attendee’s organisation or endorsement by organisation leaders [68].

Collective efficacy did not change immediately after the intervention but showed a slight increase at three-month follow-up. Descriptively, those whose intention to act increased were less likely to report improvement in collective efficacy immediately after the intervention, although this did not reach traditional levels of significance (*p* = 0.11) and the CIs crossed 0. This potentially suggests that early intention to act may have been driven more by individual agency than belief in group action, perhaps because participants in the intervention sessions did not yet know one another well, or feel confident they could act together effectively as a group [69]. The modest delayed improvement in collective efficacy may therefore reflect the time needed for participants to discuss ideas with colleagues, apply ideas within their organisational context, and build confidence in their ability to act as a team. Our qualitative data supports this interpretation; several interviewees described how following the intervention they formed new networks of like-minded colleagues, thereby discovering unexpected expertise within their organisations, and using those in-group networks to prioritise health. This underscores the greater persuasive power of peers in the in-group for motivating action, where people are consistently more likely to adopt behaviours modelled by those they see as being part of their own group [70, 71].

Consistent with our quantitative finding that participants with higher baseline intention were less likely to increase intention to act, interviewees who already prioritised health said the workshop affirmed their beliefs and prompted reflection rather than change. In contrast, those whose perspectives shifted attributed this to presentation data, peer discussions and industry partner examples that challenged assumptions about who in the sector can act on health. Although group norms did not change at three-months, these patterns are consistent with extensive evidence that social influence and normative feedback showing peers taking action can change attitudes and behaviour [71–75]. We explored potential reasons for increases and decreases in intention to act with the interviewees at T3. Although no interviewees reported a drop in intention themselves, they suggested that any observed decreases could stem from participants having an issue with content or delivery of the workshop, external constraints such as time and cost of making changes, or survey fatigue. One participant suggested increases in intention could be related to increased recognition of their ability to act.

The most commonly reported action was initiating conversations with others about prioritising health. Interviewees spoke about seeking out others in their organisation who were thinking about or acting on health, thereby building networks of people prioritising health. Some drew on resources and evidence from the Changing Mindsets intervention to persuade others to prioritise health. This use of tools and resources from the intervention can be seen as evidence of resource-based power, which is power that comes from the resources that someone holds and has access to, that provide that individual with the ability to influence another’s behaviour [76, 77], providing some indication that our intervention supported this dimension of power. Participants also described taking specific steps to prioritise health, such as raising issues highlighted in the intervention up the agenda, or initiating organisational activities related to health. However, some participants discussed having the motivation to act but acknowledged they had not yet followed through. This group may need more sustained support and follow-up to translate intention into lasting behaviour change.

### Strengths and limitations

The strength of our study is in the robust methods used to develop, optimise and evaluate our intervention. The team developed a novel theoretical framework that provided potential psycho-social influences of increased intention to act on health, which guided the design of the intervention. The intervention was co-produced with academic experts and private sector partners, and developed following the Person-Based Approach and MRC guidance [43, 45]. We used an iterative three-stage process that included synthesis of the theoretical framework and evidence, behavioural analysis, and optimisation. The intervention delivery was refined following the early events through review of participant feedback, field notes and website analytics. Adaptations were made to the intervention to improve relevance, clarity, and accessibility of the workshop content.

The intervention primarily targeted professionals in the private sector of urban development whose roles did not explicitly focus on health. However, we also sought to create opportunities for networking between this target group and others working across the urban development system, with the aim of increasing capacity to prioritise health and reducing cross-sector siloes. The intervention was therefore designed for an audience with varying levels of knowledge about the links between the built environment and health.

To support this, we provided content relevant to a range of actors. This included new tools and evidence from the TRUUD programme, such as a lived-experience video, the TRUUD cost–benefit tool, and findings on the role of coroner courts in ascribing cause of death to features of the built environment. The discussion component enabled participants to develop responses tailored to their own professional contexts through shared problem-solving. A supporting website offered more detailed resources for those seeking further depth.

Because health-related decisions in urban development are shaped by interactions between public-, private- and third-sector actors, influencing a wider group of stakeholders may help support the diffusion of new norms and practices across the system, even when an intervention is designed primarily for private-sector professionals.

Ultimately around three quarters of our sample were from our target group of professionals in the private sector of urban development across the three time-points. There was no evidence that being part of the target group had an impact on the effectiveness of the intervention. While we aimed to reach people whose role did not prioritise health, our findings that baseline intention to act on health was high indicates we attracted people who had a pre-existing interest in health. Those interviewed who spoke about already having an interest in health described how our resources had not only been useful for confirming their views on health, but also providing new data and tools that supported their efforts to convince others to act on health. This indicates that our intervention supported our secondary outcomes of increasing actions to prioritise health in those whose intention did not change.

We developed new surveys to measure knowledge-based power and pro-health norms. However, there was not capacity within the project’s timeframe to validate these measures prior to piloting them in the intervention surveys. Existing measures of power did not reflect the conceptualisation of power that underpinned our intervention theory. While utilising existing measures may have more accurately measured perceived levels of power, mapping existing measures of power onto our theoretical framework would not have been possible. We also used existing measures of collective efficacy, proximity, intention to act and resource-based power which had been previously validated but not within this population. As a result, we cannot be certain that these measures are functioning exactly as intended and therefore future validation of these measures would be beneficial.

The main limitation of this study is the small sample. The time to develop, implement and evaluate our intervention was shortened by delays in the project start caused by initial challenges in partnership building with industry organisations. Several large organisations were approached, with the collaboration talks taking a year and reaching an advanced stage before falling through. The delay caused by these negotiations ultimately limited the number of events where we could deliver our intervention and the time available for data collection. This resulted in a smaller sample size than we had anticipated and ultimately limited the range and complexity of the analysis we could conduct. In particular, we had intended to conduct quantitative analysis with a broader range of predictors of improvement in intention to act, and predictors of changes in intention to act over time.

The sample was drawn from attendees at selected industry events, and participation was voluntary, which may introduce selection bias and limit generalisability if those who chose to attend or engage with the intervention differ from the wider private-sector urban development workforce.

In addition to the small initial sample, substantial attrition across the three time-points further reduced the available sample for secondary outcomes. Although sociodemographic characteristics showed limited variation across time-points, dropout may still have been non-random. The combination of a small baseline sample, voluntary participation, and high attrition reduces statistical power, increases the likelihood of biased estimates, and limits the extent to which the findings can be generalised. These methodological constraints should be considered when interpreting the results, and replication in a larger sample with lower attrition is needed to strengthen confidence in the observed patterns.

We had also planned to qualitatively explore the power domain confirm-structuration, as there was not a robust existing survey measure [37]. Ultimately, we did not have enough interviews to meaningfully make theoretical inferences about this construct. The decision to run the study with a single arm, was a pragmatic decision to allow us to evaluate the intervention in a real world setting. However, the absence of a comparison group means we cannot attribute observed changes solely to the intervention. Future studies could include a control or comparator arm to strengthen causal inference.

### Impact on research and practice

Our findings indicate that of our theorised mechanisms to increase intention to act, proximity is the most promising. This has implications for other teams looking to shift mindsets as a precursor to behaviour change for intransigent issues. However, there would be benefits from exploring the impact of this intervention in a larger sample over a longer period, and adapting the intervention for other target groups to explore the transferability of the findings. The larger sample would support analysis exploring the impact of the theorised psycho-social influences of change in intention to act in the longer term, and allow robust analyses of suggestive findings we could not confirm with our limited data set.

Although improvement in intention to act was largely retained over the three-month follow-up, power and proximity demonstrated immediate improvement followed by a decline over time. This aligns with the well-known trajectory of behaviour-change interventions, in which initial gains often taper if not reinforced [78, 79]. This, combined with the intention–action gap described by some of our interviewees, suggests that future implementation of this intervention or adaptations could boost longer-term maintenance of intention to act by incorporating follow-up support. This could include additional sessions and digital prompts, such as brief webinars or automated messages by email or SMS reminders at intervals, which have been evidenced to be beneficial in supporting behaviour change in the longer term [80, 81]. Further adaptations to the intervention could include embedding the intervention within organisational routines, for example through mandatory training with the support of senior-leadership. Training respected peer champions to deliver intervention components, and gaining buy-in from senior leadership could provide modelling of pro-health messaging that may shift entrenched industry norms [71]. Existing social networks within the organisation could be leveraged to support the sense of collective efficacy by enabling participants to apply new ideas with trusted colleagues [70, 71]. Working with organisations that do not already prioritise health may avoid the ceiling effect of intention to act, as the attendees may have a lower baseline intention to act on health. A full exploration of recommended improvements to the intervention design will be provided in the process evaluation published separately. By combining multiple reinforcement prompts, leadership engagement, and peer influence, future interventions may be able to translate short-term motivational gains into sustained, organisation-wide changes in the prioritisation of health.

## Conclusions

This mixed-methods evaluation suggests that the Changing Mindsets intervention supported increases in intention to act on health among a subset of urban development professionals, particularly those with lower baseline intention and increased perceived proximity to health issues. While paired analyses indicated only a small average shift in intention immediately following the intervention, intention remained elevated at three-month follow-up among those who responded, suggesting that motivational gains were retained over time.

Perceived proximity emerged as the strongest predictor of improvement in intention to act, highlighting its potential role as a key mechanism for shifting mindsets in complex, upstream systems such as urban development. However, high baseline intention, ceiling effects, a small sample, and substantial attrition limit the strength of inferences that can be drawn about overall effectiveness.

Future research with larger samples, comparator designs, and longer follow-up is needed to confirm these findings and to test whether changes in intention translate into sustained organisational and system-level action. Embedding the intervention within organisational contexts and providing follow-up support may help strengthen and maintain effects over time.

## Supplementary Information

Supplementary Material 1.

Supplementary Material 2.

Supplementary Material 3.

Supplementary Material 4.

Supplementary Material 5.

Supplementary Material 6.

## Data Availability

The datasets generated and/or analysed during the current study are available from the corresponding author on reasonable request.

## Abbreviations

AIC: Akaike Information Criterion
BIC: Bayesian Information Criterion
β: Beta coefficient (regression)
CI: Confidence interval
χ²: Chi square statistic
HAUS: TRUUD Health Appraisal of Urban Systems cost and benefit tool
ID: Interview participant identifier
IF: Impact factor
IQR: Interquartile range
JSNA: Joint Strategic Needs Assessment
MRC: Medical Research Council
n, N: Subsample count; total sample size
NCDs: Non–communicable diseases
NHS: National Health Service
p: p value
QR: Quick Response (code)*
R² (pseudo R²): Coefficient of determination
REC: Research Ethics Committee
SD: Standard deviation
SMS: Short Message Service
TCPA: Town and Country Planning Association
TIDieR: Template for Intervention Description and Replication
T1, T2, T3: Baseline, immediate post session, and three–month follow–up time–points
TRUUD: Tackling the Root Causes Upstream of Unhealthy Urban Development
UK: United Kingdom
