# Supplementary material for: Evaluating changes and predictors of intention to act on health in urban development: a single-arm pre-post mixed-methods study of the changing mindsets intervention
Source: Arch Public Health. 2026 Feb 6;84:52. doi: 10.1186/s13690-026-01843-0 (PMC12997952; doi:10.1186/s13690-026-01843-0)
Supplement: Supplementary file 2 — Supplementary Material 2. [file 13690_2026_1843_MOESM2_ESM.docx]

Table 8: Categorisation of industry and occupation into target group

| Target Group | Industry | Occupation |
| --- | --- | --- |
| **No** | Consulting | Senior health and social impact consultant |
| **No** | Government (not UK) | Policy Planner |
| **No** | Research | Senior Researcher |
| **No** | Creative | Producer |
| **No** | Local Authority | Neighbourhood Coordinator |
| **No** | Third | Director |
| **No** | Public Health | Consultant in public health |
| **No** | Planning policy | Graduate planning officer |
| **No** | Research | Senior research fellow |
| **No** | Third | Projects and policy management |
| **No** | Think tank | Head of Policy |
| **No** | Charity Sector | Healthy placemaking |
| **No** | Health Consultancy | Business Case Author |
| **No** | Education | University professor |
| **No** | Regeneration (Local Authority) | Project manager |
| **No** | Academia/NHS | Evaluation/research |
| **No** | PR | Consultant |
| **Yes** | Planner | Town planner |
| **Yes** | Real estate/Professional services | Head of sustainability |
| **Yes** | Construction | ESG Manager |
| **Yes** | Architecture | Architect |
| **Yes** | Transport and Infrastructure | Transport Planning Director |
| **Yes** | Landscape Architecture/Professional | Landscape architect/Urban designer |
| **Yes** | Investment | CEO |
| **Yes** | Property | Chartered surveyor |
| **Yes** | Planning and Development | Chartered surveyor and town planner |
| **Yes** | Housing | Health and Research Lead |
| **Yes** | Architecture | Architect |
| **Yes** | Real Estate | Director |
| **Yes** | Planning | Associate Planner |
| **Yes** | Real Estate | Investment Associate |
| **Yes** | Construction | Architect |
| **Yes** | Engineering Consultancy | Director |
| **Yes** | Construction | Architect |
| **Yes** | Environmental Consultancy | EIA Coordinator |
| **Yes** | Construction | Head of social value |
| **Yes** | Architecture | Designer |
| **Yes** | Construction | Building Surveyor |
| **Yes** | Design | Architect/urban designer |
| **Yes** | Property | Social impact/ESG |
| **Yes** | Built Environment/Urban Development | Planner |
| **Yes** | Regeneration/Development | Regeneration Lead |
| **Yes** | Development | Civil Engineer |
| **Yes** | Manufacturing | Director |
| **Yes** | Engineering Consultancy | Mechanical Engineer |
| **Yes** | Development/Local Government | Architect/Design manager |
| **Yes** | Consultancy | Environmental Consultant |
| **Yes** | Real Estate | Assistant project manager |
| **Yes** | Housing | Director of Policy and Public Affairs |
| **Yes** | Architecture | Urban Design |
| **Yes** | Placemaking/Construction | Landscape Architect |
| **Yes** | Architectural Design | Architect, Principal |
| **Yes** | Placemaking - Social Enterprise | Chief operating officer |
| **Yes** | Development | Communications Manager |
| **Yes** | Housing | Social economic investment programme manager |
| **Yes** | BIDs | Sustainability Manager |
| **Yes** | Residential Development | Development Management Consultation |
| **Yes** | Placemaking/Built Environment | Senior cultural strategist |
| **Yes** | Built Environment | Social impact consultation |
| **Yes** | Built Environment | Consultant |
| **Yes** | Architecture | Architect |
| **Yes** | Architecture/Care + Residential | Architect |
| **Yes** | Real Estate | Development Manager |
| **Yes** | Architecture | Manager |
| **Yes** | Residential Development | Director |
| **Yes** | Housing | Membership Manager |
| **No** | Legal | Solicitor |
