# Supplementary material for: Evaluating changes and predictors of intention to act on health in urban development: a single-arm pre-post mixed-methods study of the changing mindsets intervention
Source: Arch Public Health. 2026 Feb 6;84:52. doi: 10.1186/s13690-026-01843-0 (PMC12997952; doi:10.1186/s13690-026-01843-0)
Supplement: Supplementary file 3 — Supplementary Material 3. [file 13690_2026_1843_MOESM3_ESM.docx]

Table 7: Data collected across the three timepoints

| **Concept to measure** | **Detail** | **Survey** | | | **Interview**  **(3 month follow-up)** |
| --- | --- | --- | --- | --- | --- |
|  |  | **Timepoint 1 (Immediately before the intervention)** | **Timepoint 2 (Immediately following the intervention)** | **Timepoint 3 (3 month follow-up)** |  |
| Sociodemographic | Age | ✓ |  |  |  |
| Sociodemographic | Gender | ✓ |  |  |  |
| Sociodemographic | Ethnicity | ✓ |  |  |  |
| Sociodemographic | Industry | ✓ |  |  |  |
| Sociodemographic | Occupation | ✓ |  |  |  |
| Proximity | Cognitive – Salience | ✓ | ✓ | ✓ |  |
| Proximity | Cognitive – Knowledge | ✓ | ✓ | ✓ |  |
| Proximity | Cognitive – Relevance | ✓ | ✓ | ✓ |  |
| Proximity | Emotional – Empathy | ✓ | ✓ | ✓ |  |
| Proximity | Mean proximity | ✓ | ✓ | ✓ |  |
| Collective efficacy | Empowerment | ✓ | ✓ | ✓ |  |
| Collective efficacy | Social cohesion | ✓ | ✓ | ✓ |  |
| Collective efficacy | Social control | ✓ | ✓ | ✓ |  |
| Collective efficacy | Mean collective efficacy | ✓ | ✓ | ✓ |  |
| Power | Resource-based | ✓ | ✓ | ✓ |  |
| Power | Knowledge-based | ✓ | ✓ | ✓ |  |
| Power | Confirm structuration |  |  |  | ✓ |
| Power | Mean power | ✓ | ✓ | ✓ |  |
| Intention to act | I intend to take concrete steps to do something to integrate health into my work | ✓ | ✓ | ✓ |  |
| Intention to act | I intend to get involved in groups or networks that focus on prioritising health | ✓ | ✓ | ✓ |  |
| Intention to act | I intend to encourage my colleagues to change their behaviour with regards to prioritising health | ✓ | ✓ | ✓ |  |
| Group norms | Balancing health with other priorities (Descriptive and Injunctive) | ✓ |  | ✓ |  |
| Group norms | Getting development done is the priority | ✓ |  | ✓ |  |
| Group norms | Already acting on health | ✓ |  | ✓ |  |
| Group norms | Innovations in planning can be risky | ✓ |  | ✓ |  |
| Group norms | Responsibility for pushing health should come from others | ✓ |  | ✓ |  |
| Group norms | Strongest driver is needs of the client | ✓ |  | ✓ |  |
| Group norms | Mean group norms | ✓ |  |  |  |
| Implementation- Dose, quality | I felt able to engage in the discussion |  | ✓ |  |  |
| Implementation- quality | I felt the discussion was valuable |  | ✓ |  |  |
| Implementation- quality | I feel like I have a better understanding about how to incorporate health into my work |  | ✓ |  |  |
