# Supplementary material for: Evaluating changes and predictors of intention to act on health in urban development: a single-arm pre-post mixed-methods study of the changing mindsets intervention
Source: Arch Public Health. 2026 Feb 6;84:52. doi: 10.1186/s13690-026-01843-0 (PMC12997952; doi:10.1186/s13690-026-01843-0)
Supplement: Supplementary file 4 — Supplementary Material 4. [file 13690_2026_1843_MOESM4_ESM.docx]

Table 6: Event details and participant eligibility

| **Event number** | **Description of the event where the intervention was delivered** | **Industry partner delivering session** | **Recruitment** | **Intervention session details** | **Issues in the session** | **Alterations to the session in response to this session** | **Number completing pre and post surveys/ Approximate total number of attendees in the session**  **(n/N,%)** |
| --- | --- | --- | --- | --- | --- | --- | --- |
| **1** | **In-person, one day conference to tackle the major issues facing UK towns, cities and environment, targeting people working in urban places and mixed use property, including people in the private, public and third sector.** | **1** | **The session was advertised through the event social media channels and in the programme** | **1 hour, in-person, the session was run as part a parallel session as part of the scheduled programme for the event and was paid for by TRUUD. Participants were sat in tables of 5-6 and worked on paper and pen during the discussion sessions.** | 1. **The audio did not work for the ‘lived experience video’ – so the video was skipped.** 2. **The Online voting/feedback software account would not log in so the feedback element of the discussion session was done through asking for feedback from each table (group of approximately 5-6).** | **As paper based working for the discussion and feedback worked well it was decided to use this for future event, unless we were working with larger groups or online events to ensure we reduced the possibility for technical issues with the online voting/feedback software.** | **20/34 (58%)** |
| **2** | **Combined half day in-person event with the TRUUD team developing a policy led intervention. The event aimed to bring together critical stakeholders from the public, private and third sectors on an invite-basis to discuss innovative and evidence-based ways to create healthier urban environments The half day consisted of a planery with the whole group. Followed by invite-only parallel workshops/sessions run by different TRUUD intervention teams. The final session included the chairs of each workshop who provided a summary of the session and participants were invited to ask questions. They were then asked to write an action to take forwards on a postcard that will be posted back to them in 6 months- to remind them of their action and to offer help from the TRUUD team to progress the action.** | **2** | **Participants were invited to the session from an existing list of senior decision makers in the private sector of urban development. The invites were opened up to people in the third and public sector after initial slow uptake from the target group.** | **1 hour. Invite-only parallel workshop sessions. Participants were sat in tables of 5-6 and worked on paper and pen during the discussion sessions.** | **None** | **The slides on what the industry partner’s company were doing on health were edited for clarity for future sessions (events 4 and 6)- The slides now showed the process by which the company had begun to incorporate health into their business model and then highlighted, challenges and solutions to getting the business on board with health.**  **Feedback from the first two events was that participants would like less focus on the presentation component and more time for discussion and feedback- all future sessions were planned to be 1 ½ hours to allow for this. The two discussion components were also split up, so we asked ‘What are your colleagues already doing on health?’ after the lived experience video, and ‘What can be done to raise health up the agenda’ left on the penultimate slide.** | **12/16 (75%)** |
| **3** | **In person 2 day conference with a focus on healthy and sustainable cities and communities, attendees were from the private, public and third sector. The event was hybrid online and in-person. As there was no capacity to put deligates into online rooms for discussion the decision was taken not to include online delegates in the research because the discussion component is core to the session the team were concerned that including the group who were not involved in the discussion would reduce the fidelity of function of the intervention/this group were only used for the process evaluation exploring mechanisms of action** | **1** | **The session was advertised through the event social media channels and in the programme** | **1 ½ hours, the session was run as part of a TRUUD sponsored ‘stream’ of the event where several elements of the TRUUD programme were presented. The online voting/feedback software was used for feedback due to the potential for a large number of attendees.** | **The online voting/feedback software worked but populated the wrong slide and showed the second question on the app for the first. One group answered the second question first the rest of the room completed the first one. We then cleared down the slide so people could populate it for the second question. But not many people engaged with it.**  **The feedback was given by going around each table for each question.** | **None** | **14/25 (56%)** |
| **4** | **1 ½ hour webinar co-hosted with Women in Property** | **2** | **Participants were** | **The 1 ½ hour session was the only element of the webinar. Participants were put into online rooms for discussion sections and asked to feedback either using the online voting/feedback software or in person when they were brought back into the main online room.** | **None** | **None** | **13/63 (21%)** |
| **5** | **In person 2 hour event developed in collaboration between the Housing Forum, the Changing Mindsets intervention team, and the Town and Country Planning Association (TCPA). The intervention session was run followed by a 30 minute presentation from the TCPA on their Healthy Homes pledge and a private sector developer reflecting on their experience of using the Healthy Homes Pledge.** | **1** | **The TCPA hosted the event and the Housing Forum invited mid and senior level members to the event.** | **1 ½ hours, in-person. The TRUUD team presented the intervention in the first session and the TCPA presented their Healthy Homes Pledge in the second session.** | **None** | **None** | **10/10 (100%)** |
| **6** | **1 ½ hour webinar hosted by TRUUD only delivering the intervention** | **2** | **There was limited advertising for this as it was at a similar time to event 5 and we did not want take participants from event 5. The TRUUD communication lead** | **1 ½ hours, online** | **None** | **None** | **0/8 (0%)** |
