# Supplementary material for: Evaluating changes and predictors of intention to act on health in urban development: a single-arm pre-post mixed-methods study of the changing mindsets intervention
Source: Arch Public Health. 2026 Feb 6;84:52. doi: 10.1186/s13690-026-01843-0 (PMC12997952; doi:10.1186/s13690-026-01843-0)
Supplement: Supplementary file 5 — Supplementary Material 5. [file 13690_2026_1843_MOESM5_ESM.docx]

Topic guide for the intervention evaluation

Introduction

- Thanks, introduce self and re-state the purpose of the interview- to explore how health is viewed by those working in urban planning and development.
- Thank for receiving the consent form/ if consent has not been received- go over verbally and ask to send completed form after the interview.
- Tell them we would be pleased to get criticism as it’s what helps us make the intervention better
- Check happy that the conversation is audio recorded
- Any questions before we start?

-----------------------------------------------------------------------------------------------------

Views on the intervention (5 minutes)

1. What did you think of the workshop?

- Prompt: anything done well, anything that could be improved?
- What did you think of the: presenter, video, discussion, examples from the industry insider

1. Did you use the website- if so what did you think of it?

Mindset change (5 mins)

1. Since the workshop, do you find yourself thinking differently about health, particularly in your work? To what do you attribute this change?
2. Prompt the workshop, other places you have heard about health, others acting on health

Actions taken? (10 mins)

1. Thinking back to the workshop and the action you planned to take, were you able to progress with the action(s) you identified?
2. Probe for any actions taken– asking others for help, building networks with other interested people, researching how to integrate health, visiting the Changing Mindsets resources page, planning a pilot study etc.
3. Probe for challenges, facilitators
4. Any further actions they plan to take?
5. Our data from the events is showing that for some people their intention to take concrete steps to prioritise health in their work decreased while for others it has increased. Do you have any insights into what might explain the decrease? The increase?

Risk and benefits (5 minutes)

1. To what extent are you thinking about risks and opportunities (benefits) when you are thinking about or prioritising health in your work?

Ripple effects (10 mins)

1. In what ways do you think you have influenced other people to start thinking more about health? Prompts- personal life, colleagues, senior management?
2. Have any actions come from these conversations or connections with others since the workshop?

Confirm structuration and norms (5 minutes)

1. What has the reception been from your colleagues (bosses/subordinates) to the introduction of health into discussions and work outputs? How have they supported/ blocked these efforts?
2. If they haven’t brought it up with colleagues- why?

TRUUD programme wide evaluation Question (5 minutes)

1. In TRUUD we are also working with the public sector to try and shape healthier urban development. How do you think that government could incentivise you to think differently about health in your work? In what ways? For example, through guidance, regulations, or the law.

Cross intervention prompts (if hear these terms, prompt for where interviewee engaged with/ heard about it):

1. Health impact assessment
2. Frome Gateway
3. Regeneration Framework
4. Bristol City Council
