# Supplementary material for: Evaluating changes and predictors of intention to act on health in urban development: a single-arm pre-post mixed-methods study of the changing mindsets intervention
Source: Arch Public Health. 2026 Feb 6;84:52. doi: 10.1186/s13690-026-01843-0 (PMC12997952; doi:10.1186/s13690-026-01843-0)
Supplement: Supplementary file 6 — Supplementary Material 6. [file 13690_2026_1843_MOESM6_ESM.pdf]

Tackling Root causes Upstream of Unhealthy Urban Development

WELCOME!

This presentation is part of the TRUUD research project. Therefore, we will start the presentation at five minutes after the allotted session time because ...

We would be delighted if you could complete the consent form and survey in front of you before the presentation begins. It will take no more than 5 minutes. A second survey is also on your desk to be completed at the end.

Tackling Root Causes Upstream of Unhealthy Urban Development

UK Prevention Research Partnership

Opportunities to prioritise health impact in urban design and development

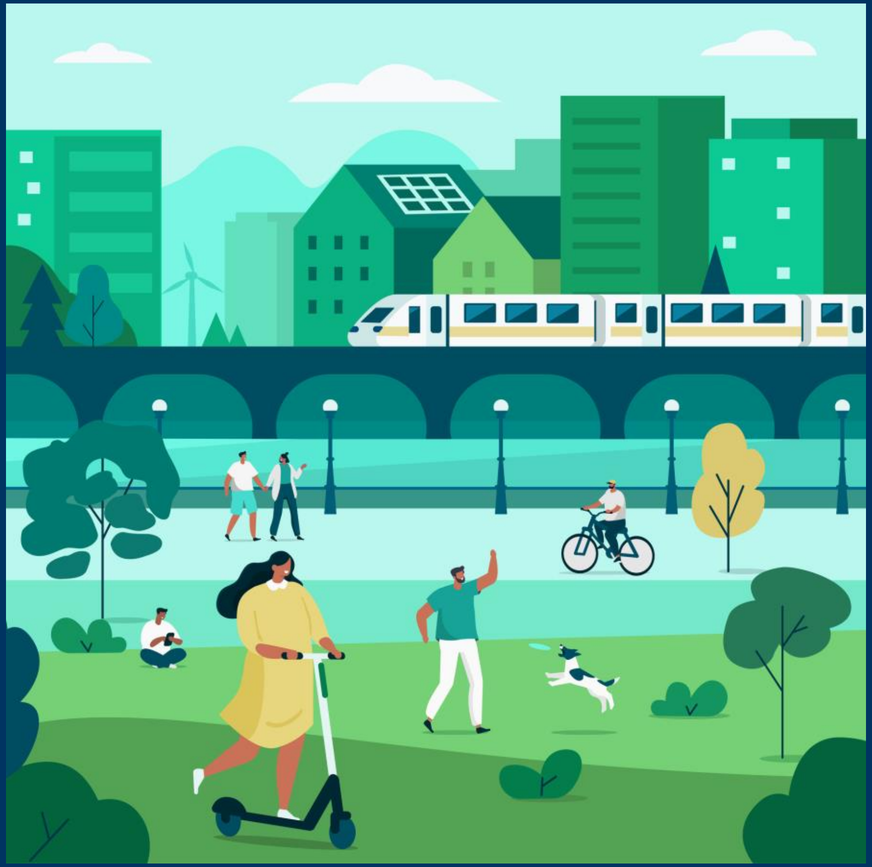

Session content

- The influence of the urban environment on health
- Risks of (not) prioritising health in development
- Benefits of prioritising health – to you and society
- Examples of what others are doing on health
- Whats next- signposting to additional resources

The problem – life expectancy is falling

DANDARA LIVING OXFORD TRUUD

Figure 1: Life expectancy in the UK in 2020 to 2022 fell to approximately the level of a decade earlier (2010 to 2012) for males and for females

Life expectancy at birth for males and females, UK, between 1980 to 1982 and 2020 to 2022

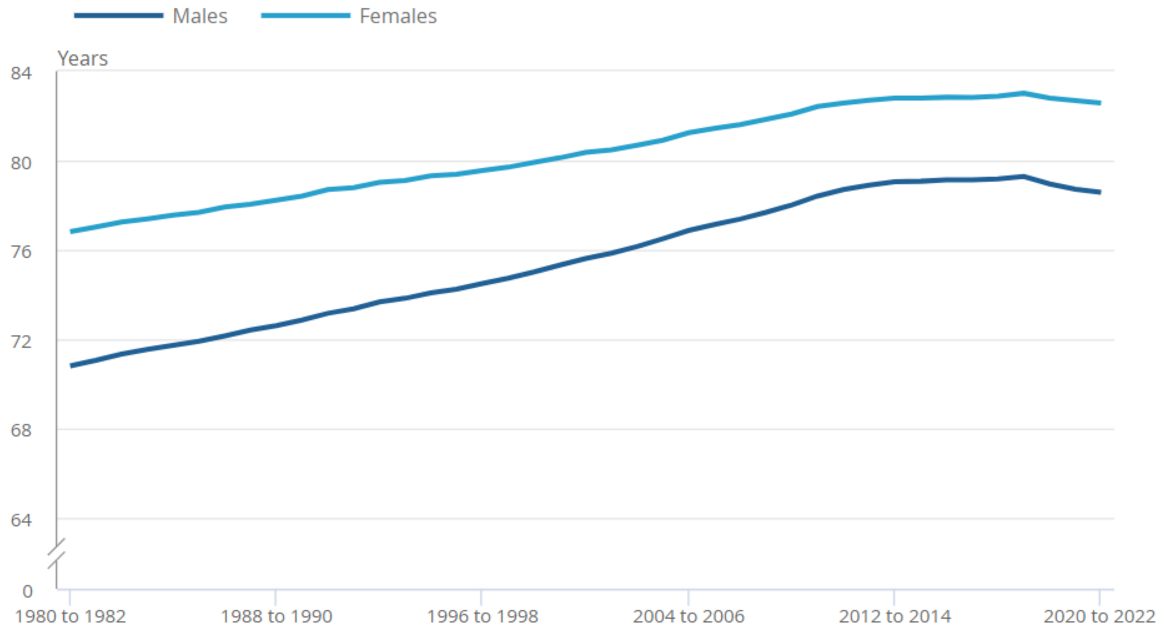

Source: National life tables - life expectancy in the UK: 2020 to 2022 from the Office for National Statistics

- Non-communicable diseases (e.g. obesity, heart failure, cancer, and mental ill-health) cause an estimated 89% of deaths

- Medical care is the largest cost facing UK taxpayers - almost 10% (c.£200bn) of total annual Government spending

- Climate and ecological impacts (linked to Non-Communicable Diseases), likely to dwarf these costs.

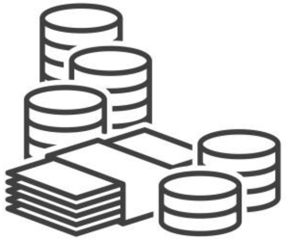

We all agree - healthy environments emerge from careful placemaking and keeping

DANDARA LIVING OXFORD TRUUD

224 professionals agree:

Health is important

Increase as priority

Increasingly colleagues doing more

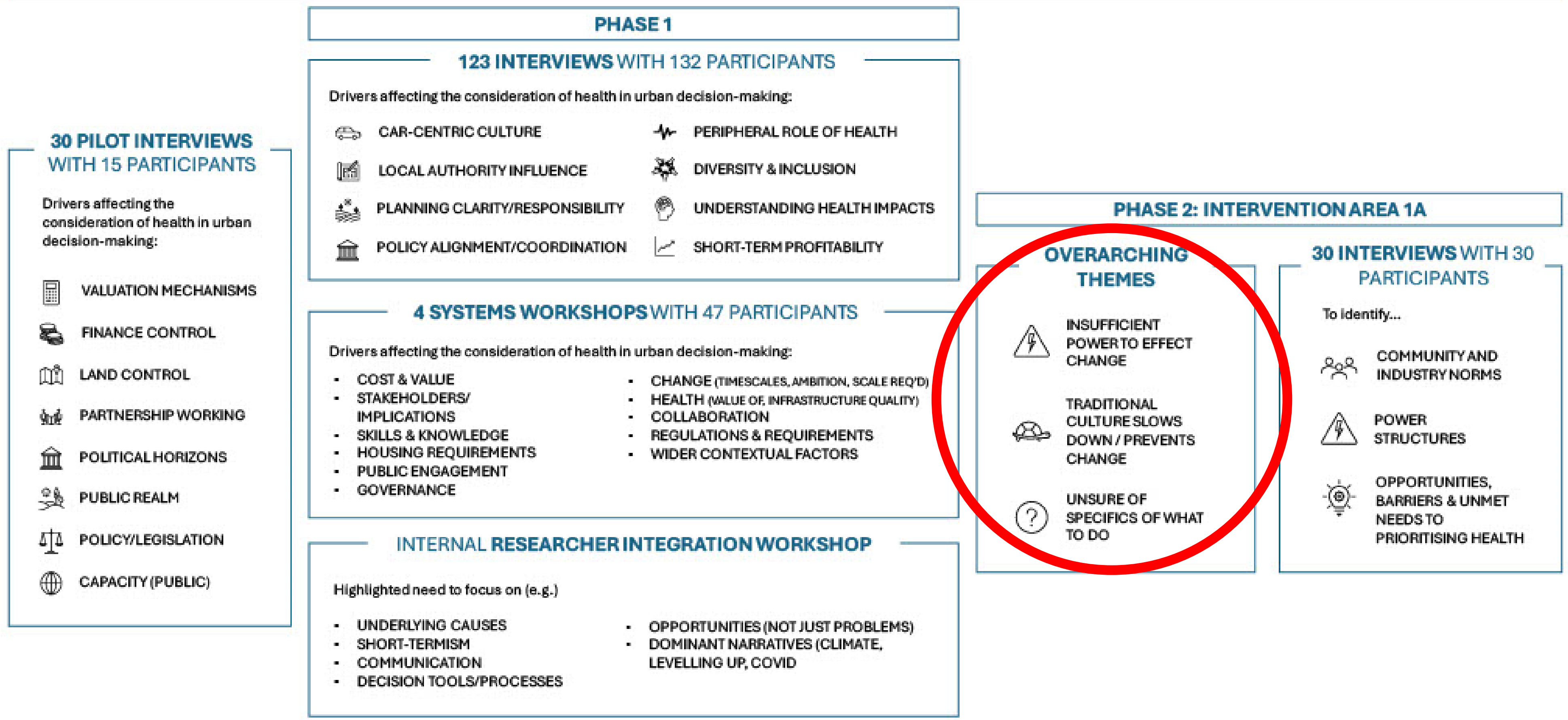

What are the conditions for health?

DANDARA LIVING OXFORD TRUUD

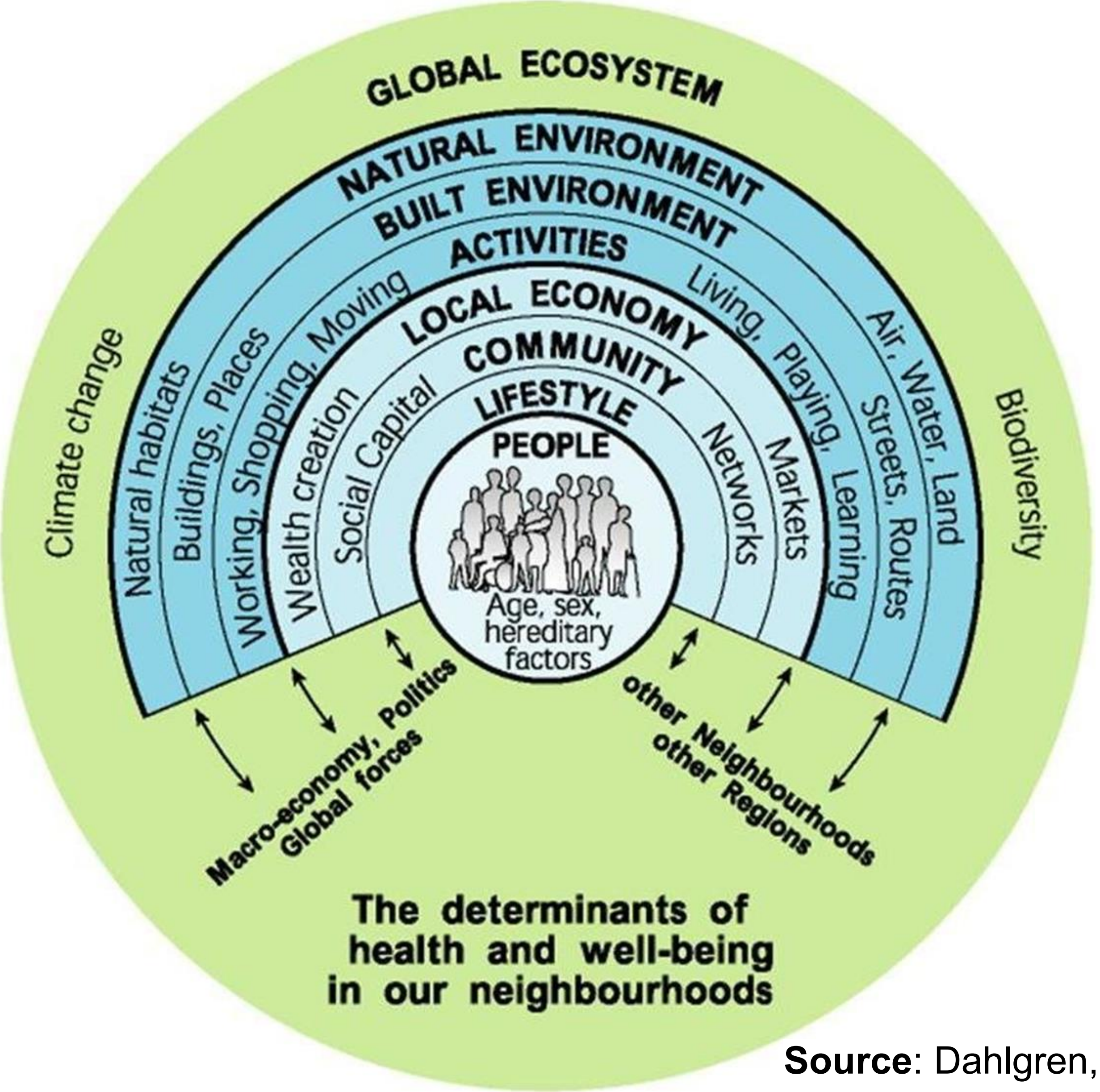

Wider determinants of health - diverse range of social, economic and environmental factors which influence people’s mental and physical health.

Source: Dahlgren, Whitehead, Barton & Grant

We know how health is being affected by the urban environment

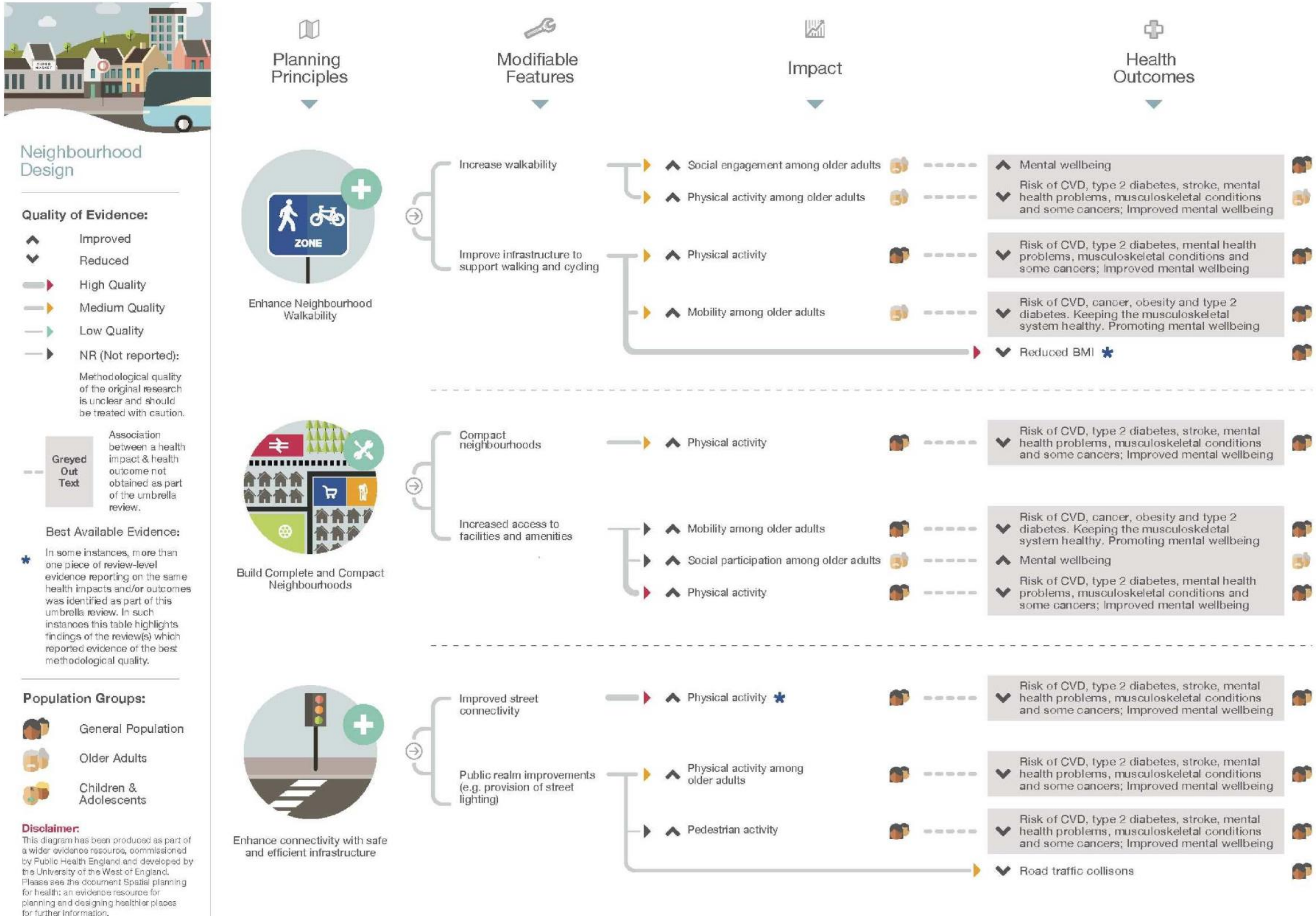

Source: Spatial Planning for Health: an evidence resource for planning and designing healthier places

We know how health is being affected by the urban environment

DANDARA LIVING | OXFORD | TRUUD

Example sub-category – ('walkability') - multiple pathways.

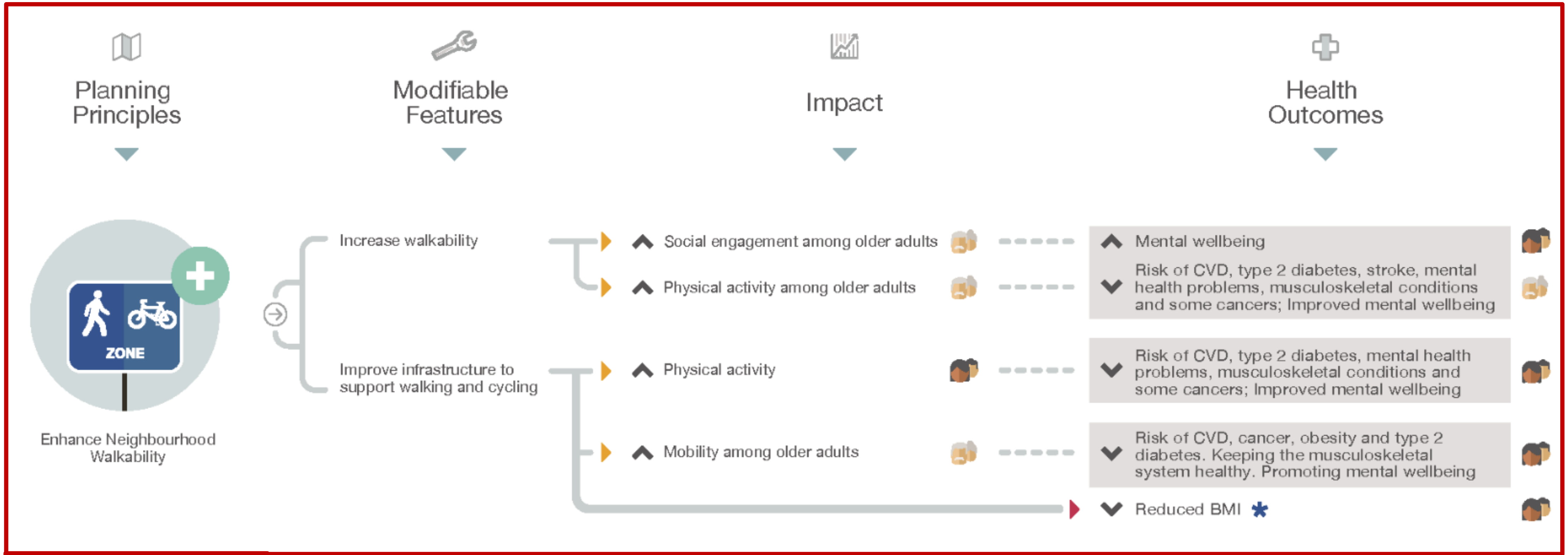

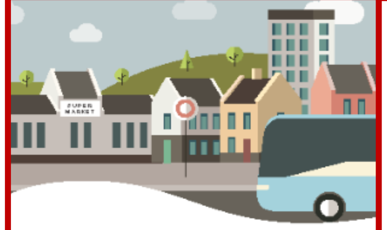
Neighbourhood Design

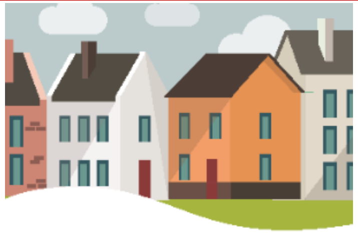
Housing

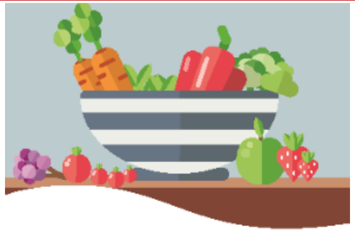
Healthier Foods

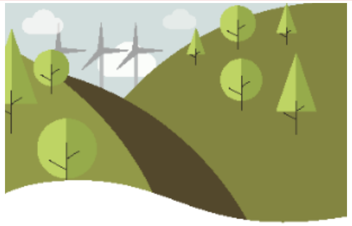
Natural & Sustainable Environments

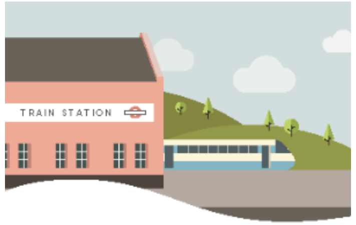
Transport

Source: Spatial Planning for Health: an evidence resource for planning and designing healthier places

How is health linked to social value/impact?

DANDARA LIVING oxford TRUUD

- Social impact of developments is increasingly being expected and measured
- While there is yet little consensus on a definition, social value can be organised under three broad themes.

SUMMARY OF OUTCOMES

JOBS AND ECONOMIC GROWTH	HEALTH, WELLBEING AND THE ENVIRONMENT	STRENGTH OF COMMUNITY
Decent jobs for local people, including hard to reach groups	Good accessibility and sustainable transportation	Strong local ownership of the development
Local people with the right skills for long-term employment	Resilient buildings and infrastructure	Existing social fabric is protected from disruption
School leavers with career aspirations of the industry	High-quality public and green spaces	The new community is well integrated into the surrounding area
The local supply chain is supported and grown	Good mental health	Thriving social networks
Future residents have comfortable homes which are affordable to operate	Good physical health	Vibrant diversity of building uses and tenures
Thriving local businesses	Healthy local air quality	Strong local identity and distinctive character
	Limit resource use and waste	

The following sections map the opportunities associated with these outcomes against the development lifecycle:

Investment → Planning → Design → Construction → Operation

Source: Social value in development: An introductory guide for local authorities and development teams (March 2018): UKGBC

We don’t all face these problems equally

DANDARA LIVING OXFORD TRUUD

Air quality and noise pollution…

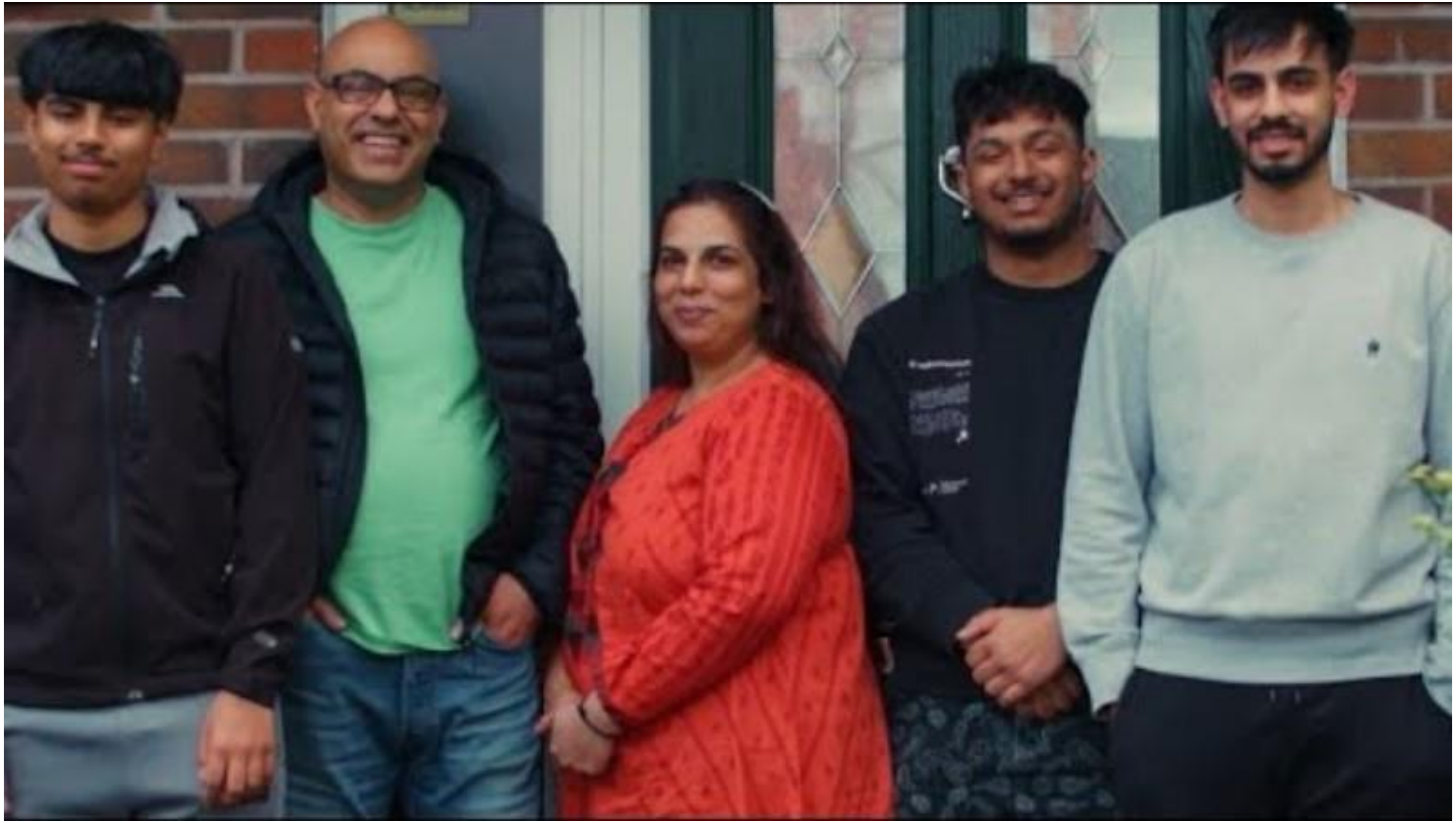

Source: TRUUD original videos - scan QR code on postcard for this and other videos of the lived experience of urban environments

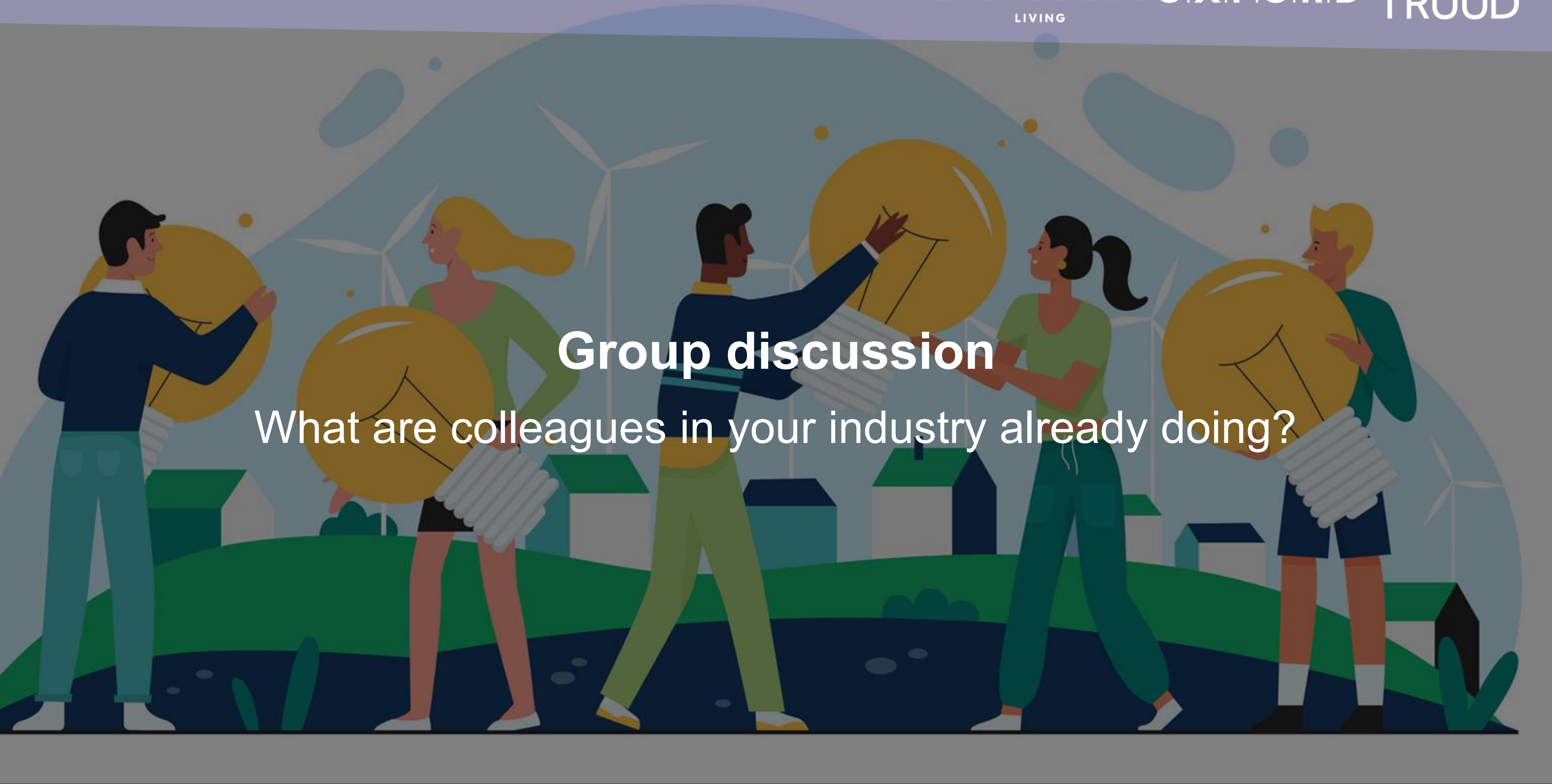

Group discussion

What are colleagues in your industry already doing?

Risks of not doing enough

- Prioritising health can feel risky
- But…. increasing risk of not priorisiting health:
  - Changes in expectations in the:
    - Marketplace
    - Planning process
  - Financial/operation risks of not doing something
  - Changes in the legal system:
    - Role of Coroners' courts
    - European Court of Human Rights '*inaction on climate change violated human rights*'

Death of two-year-old from mould in flat a 'defining moment', says coroner

Awaab Ishak died in 2020, eight days after his second birthday, following 'chronic exposure' in Rochdale

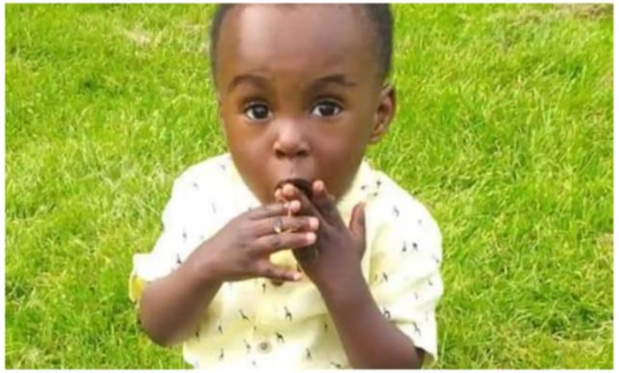

Awaab Ishak. A number of things went wrong in the case, some of them contributing to the boy's death, the coroner said. Photograph: Family handout/PA

Illegal levels of air pollution linked to child's death

3 July 2018

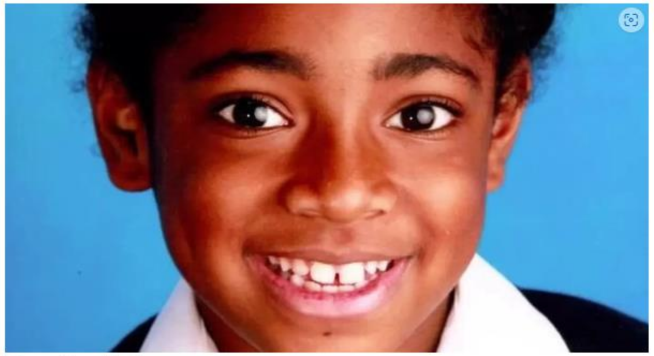

Ella wasn't born with asthma

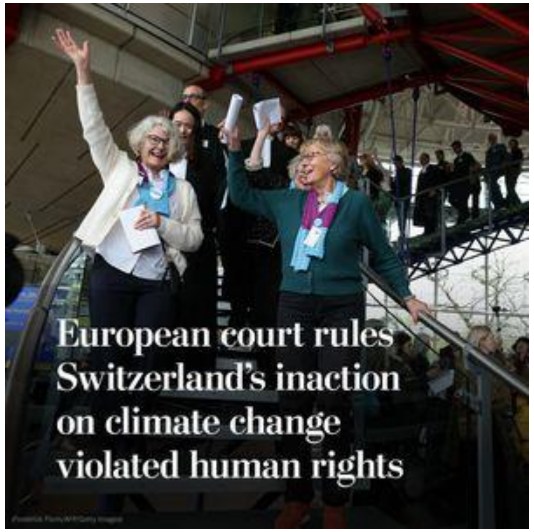

Benefits of integrating health….to you

DANDARA LIVING OXFORD TRUUD

- Reduce risks
- Prepared for market changes
- Prepared to comply with all new policies/ legislation
- Viewed as market leader leading to partner of preference
- Viewed positively by stakeholders
- Early adopters often shape policy & have favourable outcomes in government processes

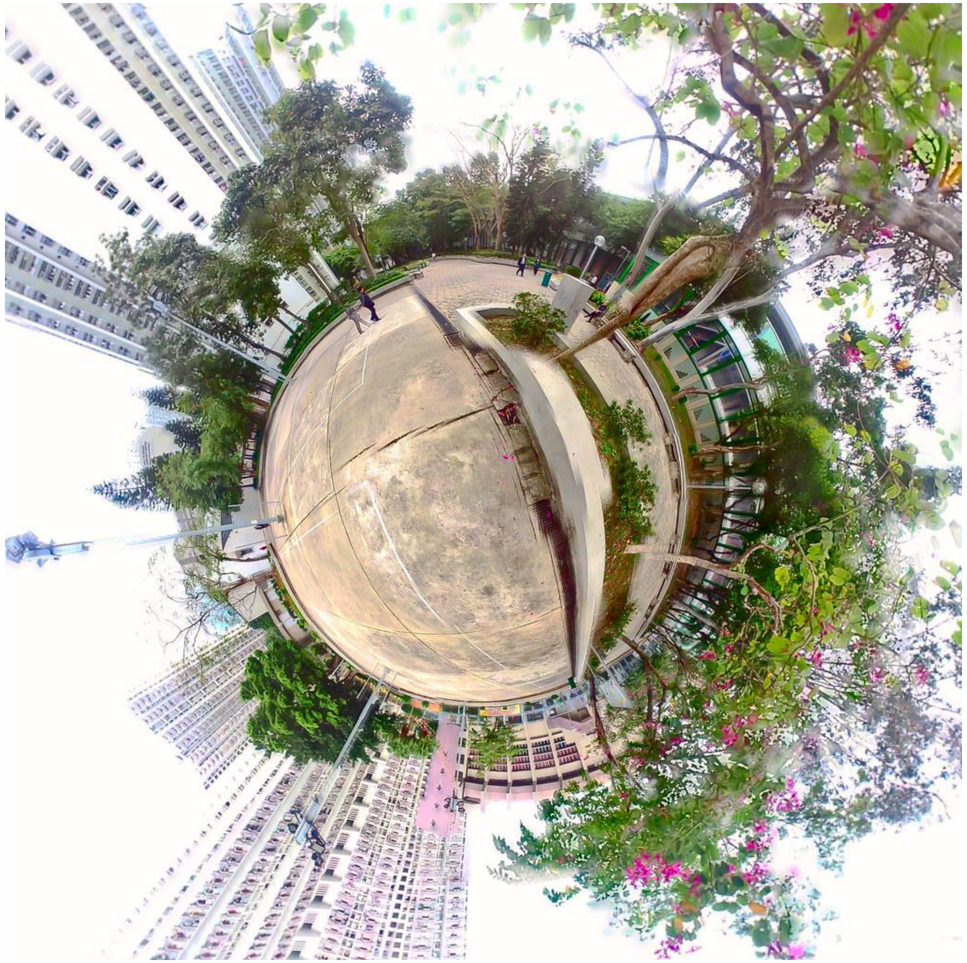

Little Planet w/ purple orchids and green leaves, Hong Kong Public Housing Panoramic Forms

Benefits to society, example: Greenspace using HAUS data

DANDARA LIVING OXFORD TRUUD

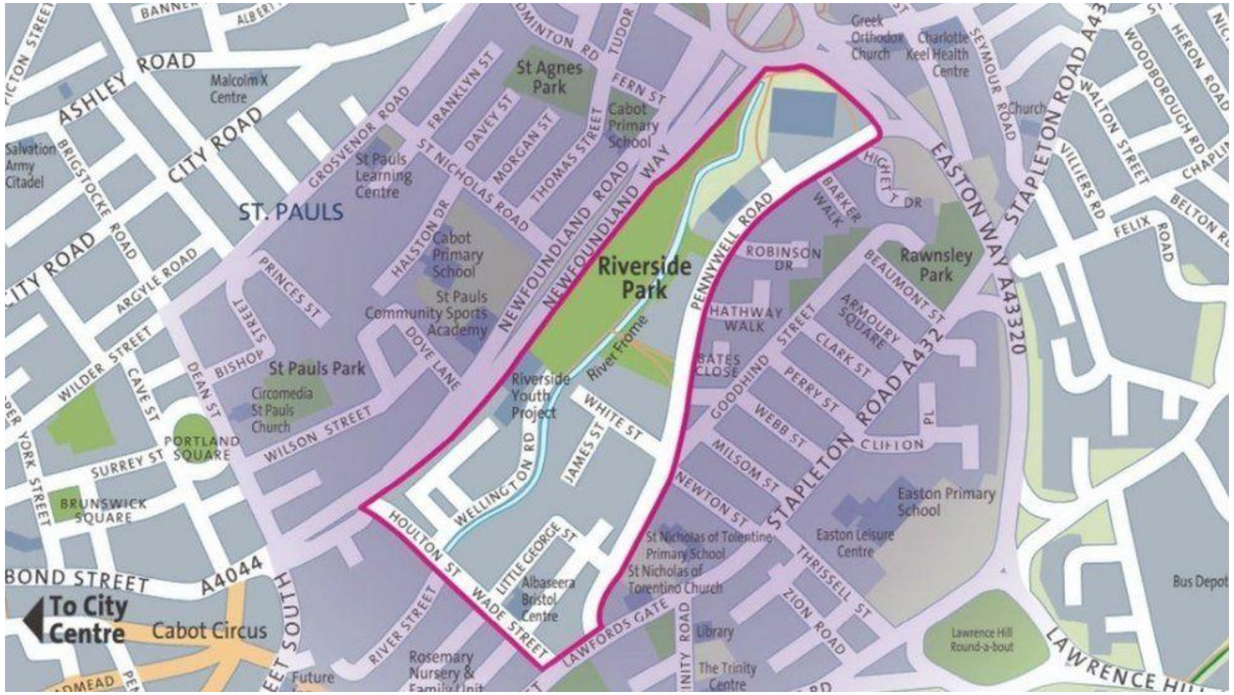

Environment change (intervention, asset or hazard)	Health outcome	Baseline cases[a]	S1 cases[b]	S2 cases[c]	S3 cases[d]
NDVI increase	Cancer (mouth and throat)	-	−0.23	−0.23	−0.23
NDVI increase	Respiratory (asthma)	-	-	8	8
NDVI increase	Weight gain	-	-	−41	−41
Proximity to green space	Activity	2,632	2,632	2,632	2,632
Proximity to green space	Mental health	125	125	125	125
Proximity to green space	Respiratory (asthma)	5	5	5	5
Proximity to large, attractive, open space	Activity	1,432	1,432	1,432	1,432
Quality of green space (pleasantness)	Life satisfaction	125	125	125	125
Quality of green space (safety)	Life satisfaction	125	125	125	125
Size of public open spaces	Diabetes	-	-	−10	-
Park improvements	Park use	-	171	171	171
Park use	Diabetes	−3	−3	−3	−3
Park use	Weight gain	−127	−139	−139	−139

[a]Baseline scenario: Population 9,241, all other conditions as present day.
[b]Scenario 1: Population 9,241, Policy Compliant.
[c]Scenario 2: Population 9,241, 1Ha additional green space in single unit.
[d]Scenario 3: Population 9,241, 1Ha additional green space dispersed across site.
Red highlighted text denotes deterioration in health. Green text indicates improvement in health.

NDVI: Normalised Difference Vegetation Index

- *Green space unlocks a wide range of health benefits*
- *Additional park of 1ha. = (potentially) 10 fewer cases of diabetes (from baseline of 41)*
    - *Over 25 years = health savings of £22.7 million*

The HAUS model is based on:

- Searches of 8,000 medical studies
- 14,000 valuation studies
- 450 primary studies
- Over 200 impact pathways

Source: Eaton E, Hunt A and Black D (2023) Developing and testing an environmental economics approach to the valuation and application of urban health externalities. Front. Public Health.

What are we already doing (on health)?

DANDARA LIVING OXFORD TRUUD

- Increasingly important topic – health inextricably linked to productivity (occupier demand)
- No single focus on health, but a broader approach with pockets of best practice
- For example:
  - 98% of buildings have a healthy food offering
  - WELL / Fitwel certified buildings globally (after covid we registered 37 office under the WELL H&S rating)
  - Healthy materials checklist for all new developments
  - Asset specific initiatives e.g. residential ceiling fans to mitigate overheating

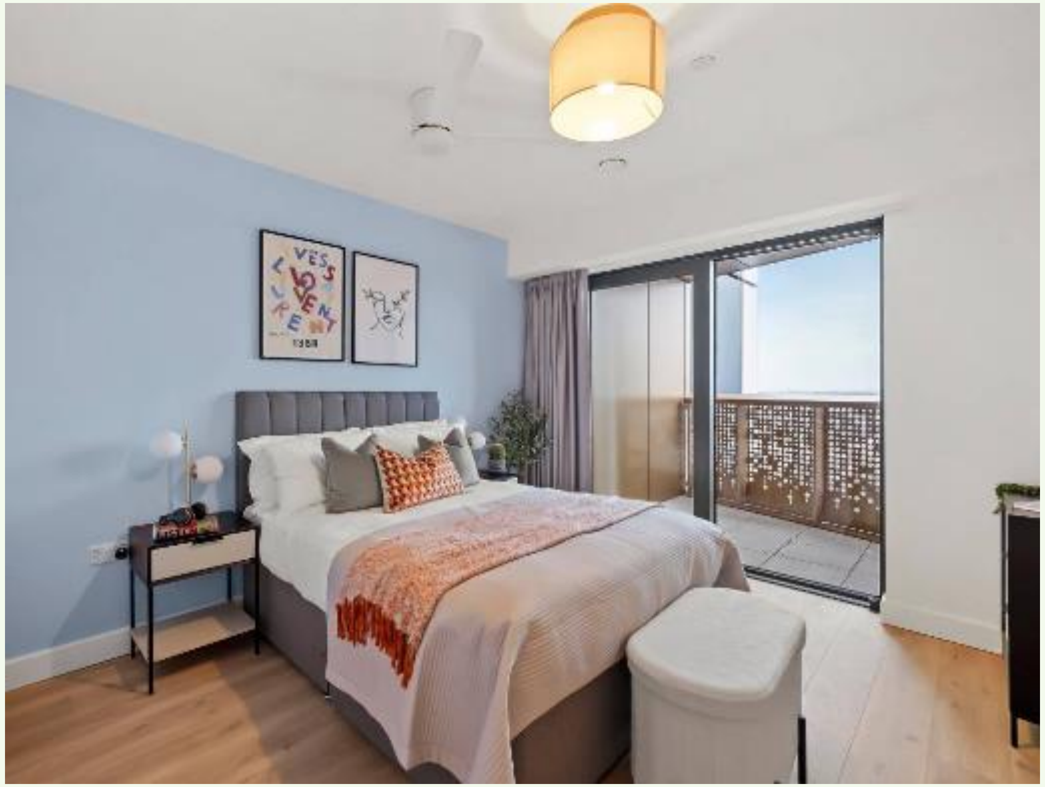

What are others doing on health?

DANDARA LIVING OXFORD TRUUD

- More natural materials use
- E.g. The Black and White Building
  - TOG / Waugh Thistleton Architects
- 45k sqft new build mass timber office in London
- 410 kgCO2e/m2
- Daylight, acoustics, temperature, biophilic design

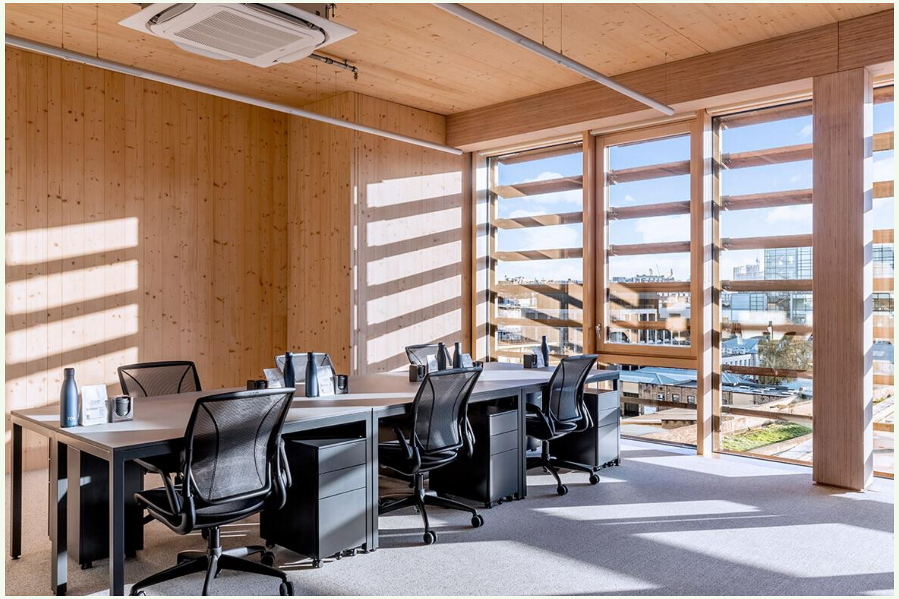

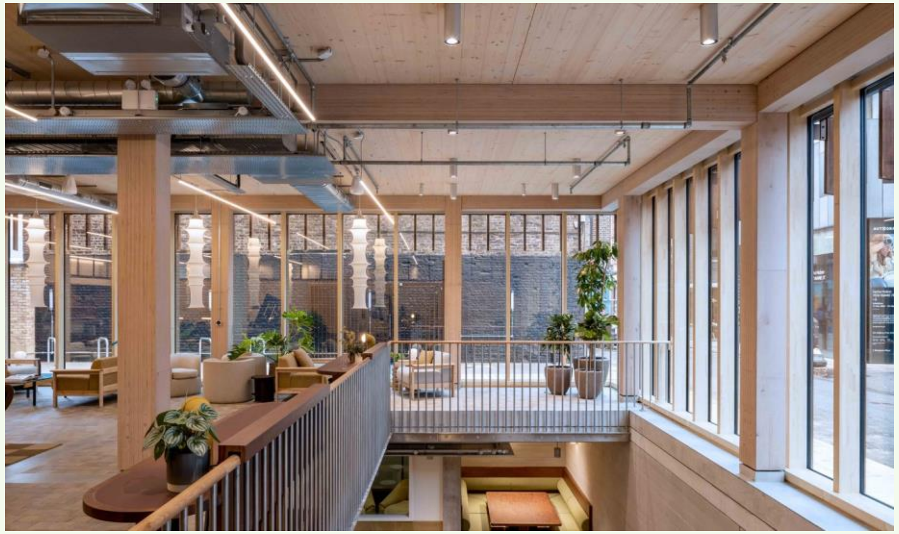

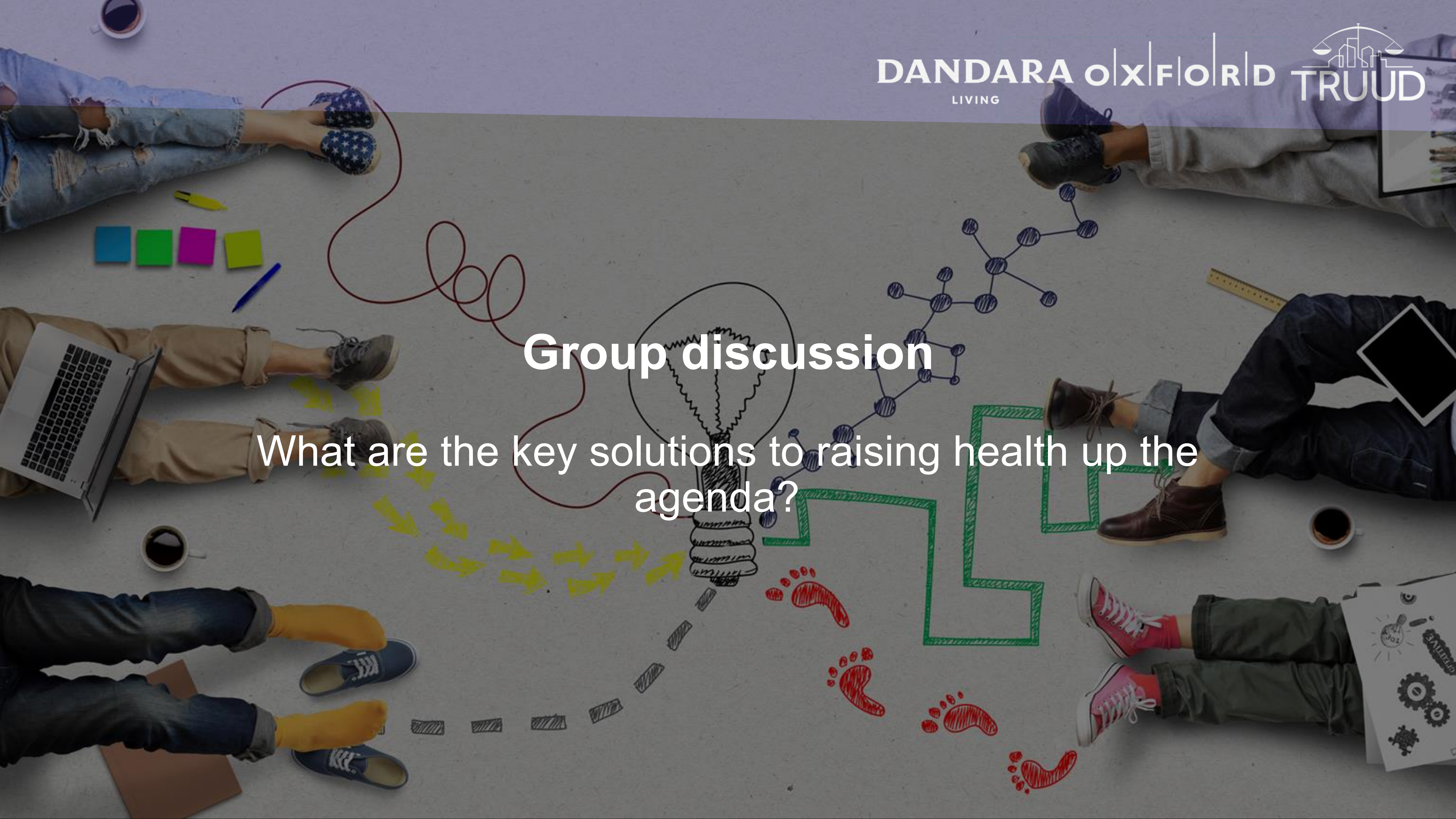

Group discussion

What are the key solutions to raising health up the agenda?

So….what action can I take today?

DANDARA LIVING OXFORD TRUUD

- Connect with others in the room (postcards)
- Commit to one action (postcards)
  - Talk to someone about how to get started
  - Join networks (check out TRUUD resource page for a list)
  - Find out what is happening in your organisation

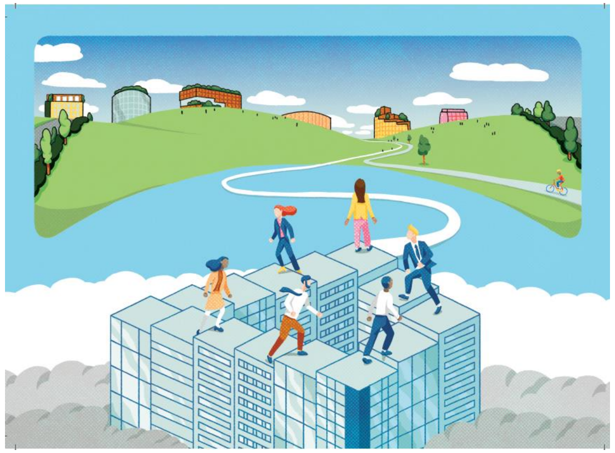

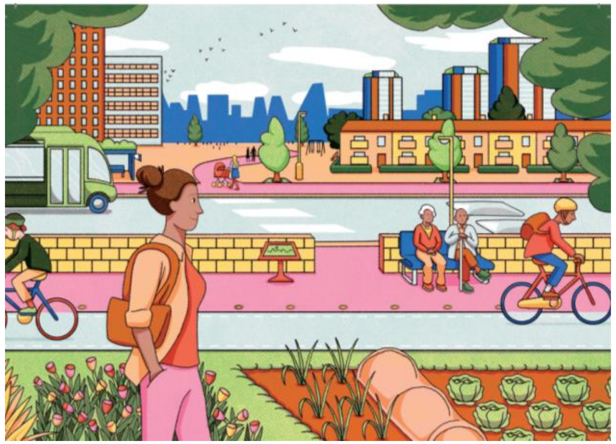

Visit our TRUUD resources page

TRUUD

Scan the QR code to access TRUUD webpage or find it at: www.truud.ac.uk/cmi-resource

Tackling Root causes Upstream of Unhealthy Urban Development

TRUUD

Contact

truud.ac.uk

Email: **truud@bristol.ac.uk**

@ResearchTruud

University Consortium

City/Combined Authority Partners

Professional Membership Partners

Research Funders

Acknowledgements

This work was supported by the UK Prevention Research Partnership, an initiative funded by UK Research and Innovation Councils, the Department of Health and Social Care (England) and the UK devolved administrations, and leading health research charities.
Weblink: https://ukprp.org/
